# Landscape of activating cancer mutations in FGFR kinases and their differential responses to inhibitors in clinical use

**DOI:** 10.18632/oncotarget.8132

**Published:** 2016-03-16

**Authors:** Harshnira Patani, Tom D. Bunney, Nethaji Thiyagarajan, Richard A. Norman, Derek Ogg, Jason Breed, Paul Ashford, Andrew Potterton, Mina Edwards, Sarah V. Williams, Gary S. Thomson, Camilla S.M. Pang, Margaret A. Knowles, Alexander L. Breeze, Christine Orengo, Chris Phillips, Matilda Katan

**Affiliations:** ^1^Institute of Structural and Molecular Biology, Division of Biosciences, University College London, Gower St, London WC1E 6BT, UK; ^2^Discovery Sciences, AstraZeneca, Mereside, Alderley Park, Macclesfield, Cheshire SK10 4TG, UK; ^3^Section of Experimental Oncology, Leeds Institute of Molecular Medicine, St James's University Hospital, Leeds LS9 7TF, UK; ^4^Astbury Centre for Structural Molecular Biology, Faculty of Biological Sciences, University of Leeds, Leeds LS2 9JT, UK

**Keywords:** precision medicine, cancer mutations, receptor tyrosine kinases, small molecule inhibitors, resistance

## Abstract

Frequent genetic alterations discovered in FGFRs and evidence implicating some as drivers in diverse tumors has been accompanied by rapid progress in targeting FGFRs for anticancer treatments. Wider assessment of the impact of genetic changes on the activation state and drug responses is needed to better link the genomic data and treatment options. We here apply a direct comparative and comprehensive analysis of FGFR3 kinase domain variants representing the diversity of point-mutations reported in this domain. We reinforce the importance of N540K and K650E and establish that not all highly activating mutations (for example R669G) occur at high-frequency and conversely, that some “hotspots” may not be linked to activation. Further structural characterization consolidates a mechanistic view of FGFR kinase activation and extends insights into drug binding. Importantly, using several inhibitors of particular clinical interest (AZD4547, BGJ-398, TKI258, JNJ42756493 and AP24534), we find that some activating mutations (including different replacements of the same residue) result in distinct changes in their efficacy. Considering that there is no approved inhibitor for anticancer treatments based on FGFR-targeting, this information will be immediately translatable to ongoing clinical trials.

## INTRODUCTION

Fibroblast growth factors (FGFs) and their receptors (FGFR1-4) regulate a wide range of physiological processes including embryogenesis, wound healing, inflammation and angiogenesis as well as adult tissue homeostasis [1, 2]. Importantly, aberrant FGF/FGFR signaling has been linked to several developmental syndromes and a broad range of human malignancies [3, 4]. The involvement in the pathology of many cancer types provides a strong rationale for development of effective agents for these targets; consequently, there is a large ongoing effort to develop FGFR inhibitors as anticancer treatments [5, 6].

Recent applications of deep sequencing technologies have resulted in the discovery of frequent alterations in FGFR molecules; the alterations include point-mutations, overexpression and fusion genes [7–9]. Very recent analysis of about 5000 solid tumors provided a comprehensive picture of the FGFR alterations in cancer [10], generally consistent with the data combined from individual, smaller studies reported previously (examples include [11–17]). Based on these studies it is likely that FGFR aberrations occur in about 7% of solid tumors and almost every type of malignancy examined has been associated with FGFR aberrations; most commonly affected were urothelial, breast, endometrial, squamous lung cancers and ovarian cancer. The most frequently detected aberration appears to be gene amplification, in particular amplification of FGFR1, resulting in overexpression. Rearrangements resulting in fusion proteins (the most frequent being FGFR3TACC3-fusion) have been so far observed in smaller numbers compared to amplifications and mutations. Point-mutations have been detected in all FGFRs and are likely to represent about one third of all aberrations; they appear to be more frequent in FGFR3 and FGFR2 compared with FGFR1 and FGFR4.

The field of FGFR targeting has progressed rapidly in recent years especially owing to the further evaluation of nonselective and the development of novel FGFR-selective tyrosine kinase inhibitors (TKIs), many of which are currently in clinical trials [6]. The most clinically advanced compounds are nonselective TKIs (including ponatinib, brivanib, nintedanib, lenvatinib, dovitinib and lucitanib) that also have inhibitory activity against some other RTKs (such as VEGFRs). However, accumulative side effects and a modest efficacy of some of these compounds towards FGFRs have prompted the development of selective and highly potent FGFR TKIs, including AZD4547, BGJ-398 and JNJ42756493 [18–20]. It is also becoming apparent that optimal therapeutic application of FGFR inhibitors requires knowledge of the molecular profiles of populations that will benefit most from these drugs. Data providing information about the rates and types of FGFR aberrations in a variety of cancer types, as summarized above, will no doubt help to aid design of clinical trials.

The next rate-liming step to understand the clinical implications of such abundant genomic data, however, is the wider assessment of the causative role of these alterations found in cancer; the impact on protein function, including activation state and drug responses, needs to be assessed. In particular, point mutations occur in all regions of the receptor molecules including extracellular, trans-membrane and kinase domains of FGFR1-4, suggesting diverse outcomes and mechanisms [7, 9]. Several pre-clinical studies focusing on some of the frequently observed mutations demonstrated their oncogenic potential. For example, FGFR3 extracellular S249C substitution that occurs in more than 70% of urothelial tumors harboring mutations has been shown to enhance receptor phosphorylation, downstream signaling, saturation density in urothelial cells and to induce transformation of mouse fibroblasts [21]. Furthermore, viability and proliferation of some cancer cell lines with the FGFR3 S249C substitution was compromised by FGFR inhibitors including the FGFR-specific AZD4547 compound [22]. Among FGFR kinase domain (KD) cancer mutations, another FGFR3 activating mutation, K650E, has been most extensively characterized [14, 21, 23]. However, to our knowledge, there is to date no comprehensive study in which different FGFR cancer variants have been directly compared and the impact of mutations assessed quantitatively. Furthermore, it is not clear how different primary or acquired mutations can affect responses to the emerging clinically promising inhibitors, including a number of nonselective and selective TKIs. Because all currently tested TKIs inhibit receptor kinase activity by interfering with the binding of ATP or substrates of the tyrosine kinase domain, mutations occurring within this domain are likely to be particularly relevant in this context. With these aims in mind, here we describe the effect of FGFR KD mutations on kinase activity, using a comprehensive panel, and distinct signatures of drug efficacy for different activating FGFR variants.

## RESULTS

### Reported mutations in FGFR KD

**Figure 1:**
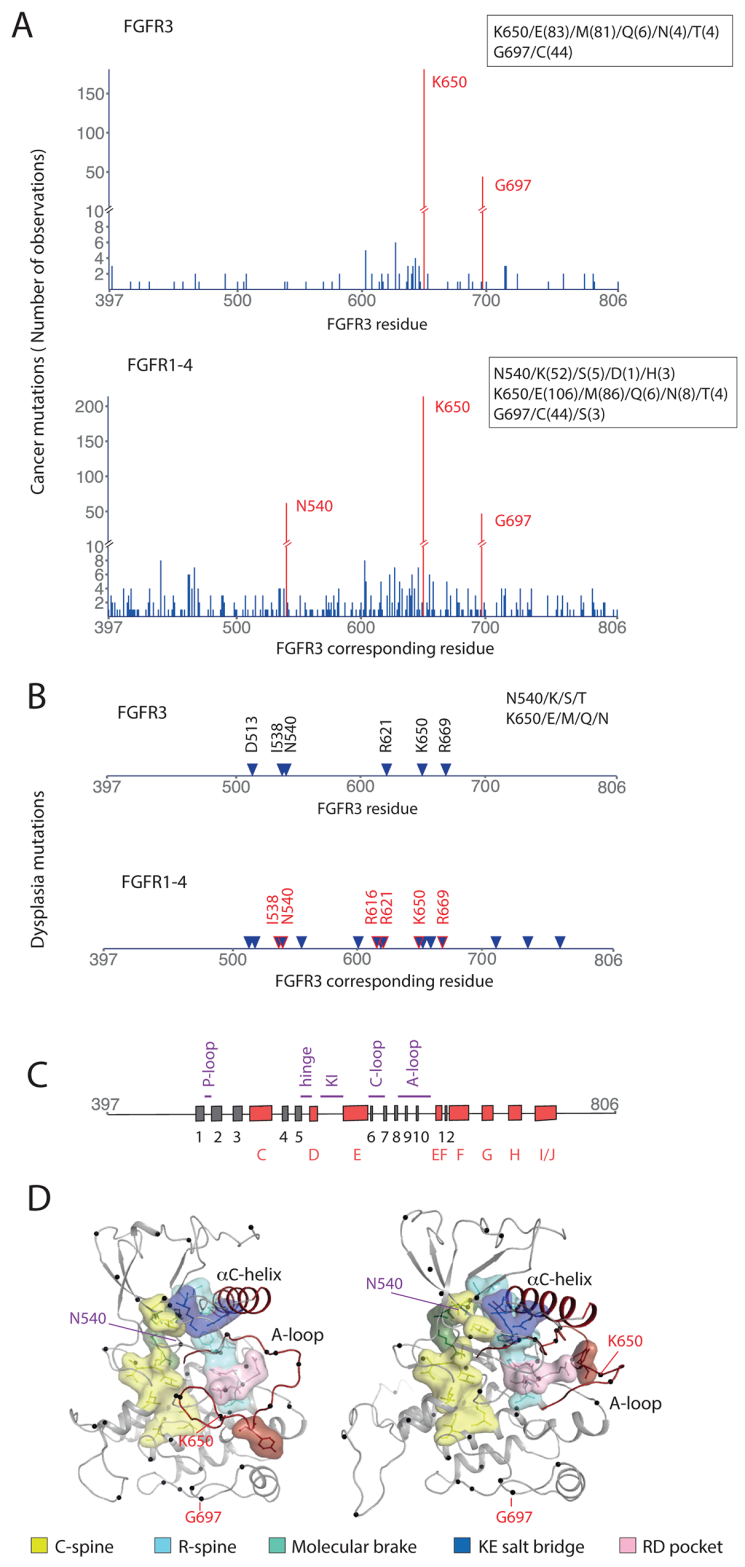
Point mutations in the intracellular region of FGFR **A.** Positions of point mutations and number of observations across different cancer types reported for FGFR3 (top) and for all FGFRs (FGFR1-4) (bottom). Hot spot positions are highlighted in red and their specific substitutions and numbers (in brackets) shown in the insets. **B.** Positions of point mutations found in skeletal dysplasia reported for FGFR3 (top) and for all FGFRs (FGFR1-4) (bottom). Specific substitutions for positions N540 and K650 in FGFR3 are shown in the inset. Positions highlighted in red are those found in both, FGFR3 in different cancers and in FGFRs in skeletal dysplasia. **C.** Secondary structure elements of the FGFR3 KD. β-strands (1-12, β11 is not present) are shown as grey boxes and α-helices (C-J) as red boxes; the key loops or disordered regions are indicated in purple. D. 3D representations of an inactive (left) and active (right) FGFR3 KD based on previously reported crystal structures of FGFR1 (PDB: 4UWY and 3GQI) and high degree of conservation (see Supplementary Figure S1). Regions implicated in transition from an inactive to an active conformation are highlighted in different colors. Hot spot positions across different cancer types reported for FGFR3 (K650 and G697) are shown in red; one additional hot spot observed in analysis of cancer mutations in all FGFRs (N540) is shown in purple.

To obtain a comprehensive insight into mutations affecting the intracellular portion (and in particular KD) of FGFRs, we compiled missense mutation data and performed multiple sequence and structure-based analyses (Figure 1, Supplementary Table S1).

The intracellular portion (residues 397-806 for FGFR3) comprises the juxtamembrane region, KD and C-terminal regions. The number of observed mutations at each residue within the intracellular portion of FGFR3 was compiled from several cancer databases (COSMIC, TCGA, ICGC and BioMuta) covering all cancer types (Figure 1A, top). Considering the high degree of similarity between FGFR1-4, the same analysis was also performed for all FGFRs and displayed using amino acid numbering for FGFR3 (Figure 1A, bottom). Two frequently mutated positions (“hotspots”) were identified in FGFR3 KD in cancer (K650 and G697) and a third (corresponding to N540 in FGFR3) was revealed only when considering all FGFRs (Figure 1A). The most frequent amino acid replacements at the position corresponding to FGFR3 K650 were E and M while others such as N, Q and T were observed less frequently. For the position corresponding to FGFR3 N540, the most frequent mutation was to K compared to S, D or H. The frequent replacement G697C was reported only for FGFR3 (Figure 1A).

It has been previously highlighted that a number of cancer mutations, in particular in FGFR2 and FGFR3, have also been described in various developmental syndromes such as bone dysplasia [4]. Positions mutated in FGFR3 in this type of dysplasia (Figure 1B top) included not only one of the hotspots observed in FGFR3 in cancer (K650) (Figure 1A top) but also the N540 hotspot position (Figure 1A bottom). N540 FGFR3 mutations in bone dysplasia reflect the overall picture of FGFR1-4 mutations in cancer with the replacement N540K being the most frequently observed [24](Figure 1B top). A distinct pattern of cancer mutations in FGFR1-4 can reflect differences in cancer types and their etiology, with FGFR3 mutations being most prevalent in a single cancer type (i.e bladder cancer) [7–9].

Comparison of positions mutated in bone dysplasia in all FGFRs with those reported for FGFR3 in cancer (Figure 1B, bottom) highlighted common residues including I538, N540, K650 and R669. With respect to the secondary structure (Figure 1C), I538 and N540 are in the αC-β4 linker, K650 in the activation (A)-loop and R669 in αEF-β12 linker. The hot spot position G697 has not been observed in bone dysplasia in any of FGFRs and is present within the αF- αG linker.

A number of crystal structures of FGFR KD in non-phosphorylated and phosphorylated forms have been reported [25]. The 3D-structures highlighted the features that undergo substantial changes and play a key role in the activation process; of particular importance is the A-loop and so called “molecular brake” (Figure 1D, Supplementary Figure S1). When positions of FGFR3 cancer mutations are highlighted in the FGFR KD structure, one hot spot (K650) and 9 other residues are present within the A-loop while R669 is in its vicinity. Residues N540 and I538 are part of the molecular brake.

In a broader context, linking genetic data with the functional outcome remains a challenge and the accuracy is such that most predictions should be seen as complementary to - rather than a replacement for - experimental evaluation [26, 27]. In addition to ranking based on observed mutation frequencies, there are a number of bioinformatics tools that can assess the possible impacts of a mutation (Supplementary Table S1A, Supplementary Figure S2). Many algorithms assume that mutations affecting protein function occur at evolutionarily conserved sites and the meta-predictor CONsensus DELeteriousness score (Condel) combines several of these [28]. More sophisticated predictors make use of available protein structures (such as SAAP, that predicts outcomes using multiple features, or 3D-clustering). Some structure-based tools allow prediction of the energetic impacts of mutations; we used FOLDX [29] to predict changes in stability for all possible FGFR3 KD mutations and ranked them. Stability differences between active and inactive KD can predict mutations expected to shift equilibrium from inactive to active conformations.

We used these tools for analysis of all reported intracellular FGFR3 cancer mutations and also included some common mutations found in other FGFRs or in bone dysplasia. Based on this, we constructed a representative panel for experimental evaluation that covers different structural elements of the KD, frequencies, cancer types and different prediction outcomes (Table 1). The panel includes about 47% of all intracellular residues reported to be mutated in FGFR3 in cancer.

**Table 1:** Panel of FGFR3 variants selected for experimental studies

Substitution in FGFR3	CancerPrimary tissue	Corresponding substitution in FGFR1, 2 or 4 in cancer	Corresponding substitution in FGFRs in dysplasia	Summary of prediction of impact based on Condel (C) and FOLDX (F)***
E466K	CNS	✓ FGFR2	-	Deleterious (C)
A500T	Pancreas	-	-	Neutral to stabilizing for the active form (F)
I538F	Hematopoietic	-	-	Very destabilizing for the inactive form and neutral to destabilizing for the active form (F)
I538V	-	✓ FGFR2	✓ FGFR3, FGFR2	Neutral (F)
N540K	-	✓ FGFR1, FGFR2, FGFR4	✓ FGFR3	Neutral to very stabilizing for the active form (F)
N540S	Urinary tract	✓ FGFR1	✓ FGFR3	Neutral (F)
V555M	Hematopoietic	-	-	Stabilizing for the active form (F)
P572A	CNS	-	-	Neutral (F)
C582F	Ovary	-	-	Neutral (F)
D617G	Aerodigestive tract	-	-	Deleterious (C) Neutral to stabilizing for the active form (F)
E627D	Urinary tract	-	-	Neutral (F)
V630M	Aerodigestive tract	-	-	Neutral (F)
G637W	Kidney	-	-	Deleterious (C) Destabilizing for the inactive form and stabilizing to very stabilizing for the active form (F)
D641G	Large Intestine	-	-	Neutral (F)
D641N	Urinary tract	✓ FGFR1	-	Neutral (F)
H643D	Urinary tract	-	-	Neutral (F)
D646Y	Urinary tract	-	-	Neutral (F)
Y647C	Lung**	-	-	Neutral (F)
K650E	Range of cancer types	✓ FGFR1, FGFR2, FGFR4	✓ FGFR3	Very stabilizing for the active form (F)
K650N	Urinary tract Large Intestine	✓ FGFR2	✓ FGFR3, FGFR2	Stabilizing for the active form (F)
N653H	Urinary tract	-		Neutral (F)
R669G	-	✓ FGFR2	✓ FGFR3, FGFR2	Neutral to very stabilizing for the active form (F)
R669Q	Large Intestine	✓ FGFR1	-	Neutral to stabilizing for the active form (F)
V677I	Large Intestine	-	-	Neutral (F)
G697C	Aerodigestive tract	-	-	Neutral (F)

* red indicates hot spot positions in FGFR3 KD, purple indicates an additional hotspot position seen for FGFR1-4 KD

** mutation has been observed in a model of resistance to EGFR inhibitors

*** FOLDX analysis for the panel was extended compared to analysis in include additional structural information

### Kinase activity of FGFR3 KD variants

The number of cancer mutations in FGFR KDs that have been comprehensively assessed for their functional impact is limited, with the most emphasis being on replacements at positions corresponding to FGFR3 K650 and mutation corresponding to FGFR3 N540K [14, 21, 23, 30, 31]. Some comparisons of mutations found in the context of bone dysplasia have also been reported [32–34]. However, very few studies compared a panel of cancer mutations or directly and quantitatively measured kinase activity; no such studies have been applied to FGFR3 KD.

**Figure 2:**
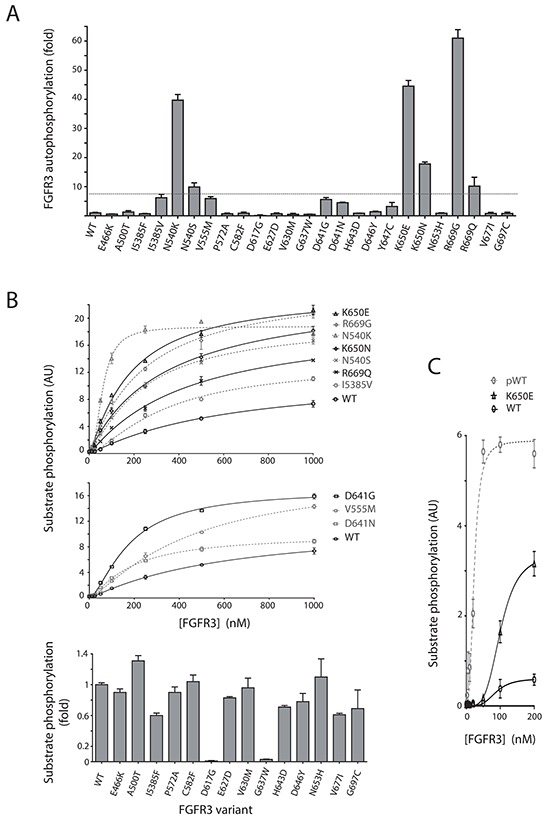
Kinase activity of selected FGFR3 variants harboring point mutations **A.** Auto-phosphorylation of purified proteins incorporating region 455-768 of FGFR3 without (WT) and with indicated substitutions. Activity of each variant is expressed as a fold change compared to the WT (WT=1). **B.** FGFR3 proteins described in A were analyzed for substrate (poly Glu-Tyr) phosphorylation. Activities of highly activating variants (top) and activating variants (middle) are shown as a function of increasing FGFR3 concentrations; the activity of other variants (bottom) is expressed as a fold change compared to the WT. **C.** Comparison of non-phosphorylated (WT), phosphorylated (pWT) and K650E FGFR3 proteins in a substrate phosphorylation assay.

Isolated FGFR KDs undergo auto-phosphorylation on several tyrosine residues and this property correlates well with the kinase activity towards natural and synthetic substrates. We used purified proteins of 26 FGFR3 KD variants to directly compare the impact of different mutations on FGFR3 KD auto-phosphorylation (Figure 2A). The most frequent mutations at the hotspot positions, K650E and N540K, resulted in a large increase (up to 45-fold) in auto-phosphorylation. Interestingly, at both positions, the mutations observed less frequently, K650N and N540S, were also less activating. However, surprisingly, the most activating mutation in this assay was a non-hotspot mutation R669G. Another replacement at this position, R669Q, also resulted in an increase of auto-phosphorylation. The least activating among variants that increased FGFR3 KD auto-phosphorylation more than 7-fold was the I538V mutation. Results from further analysis of these activating variants in a substrate phosphorylation assay (Figure 2B, top panel) were generally consistent with the auto-phosphorylation data (Figure 2A).

Several other mutations, including V555M, D641G and D641N resulted in an increase of auto-phosphorylation up to 7-fold (Figure 2A) and a similar increase in substrate phosphorylation (Figure 2B, middle panel). The V555M mutation, unlike other mutations in this panel, is an acquired resistance mutation to an FGFR inhibitor (AZ12908010) where the gatekeeper residue, V555, is replaced by a larger side-chain residue [22]. Gatekeeper mutations have been described in many kinases and several studies have shown an associated increase in kinase activity [35–37].

Twelve out of 26 analyzed mutations had very little or no effect on FGFR3 KD activity (Figure 2A and 2B, bottom panel). The number of observations in cancer for most of these mutations is low with the exception of G697C that represents one of the hotspots (Figure 1A).

Two mutations, D617G and G637W, completely abolished kinase activity (Figure 2A and 2B, bottom panel). Both residues are strongly conserved among protein kinases and some of the replacements of these residues in various kinases have been tested and shown to result in inactivation [38, 39]. D617 is the key catalytic residue and part of the HRD motif in the C-loop, while G637 resides in the A-loop as a part of the DFG motif. The DFG motif is surprisingly frequently mutated in the cancer kinome [38]. However, a G to W replacement has not been observed and tested previously and, as shown in Figure 2B (bottom panel), also results in kinase inactivation. In general, the importance of these inactivating mutations for cancer development remains unclear and specifically for FGFRs it could be linked to their suggested, context-dependent tumour protective functions [40].

From these direct measurements of kinase activity it seems that a considerable number of mutations reported so far result in kinase activation to some degree and that replacements that cause activation are not limited to hotspot positions (Figures 2A and 2B). However, the comparison of a highly activating K650E and phosphorylated WT FGFR3 KD suggests that even these mutations do not fully convert mutated molecules to their active conformations (Figure 2C).

**Figure 3:**
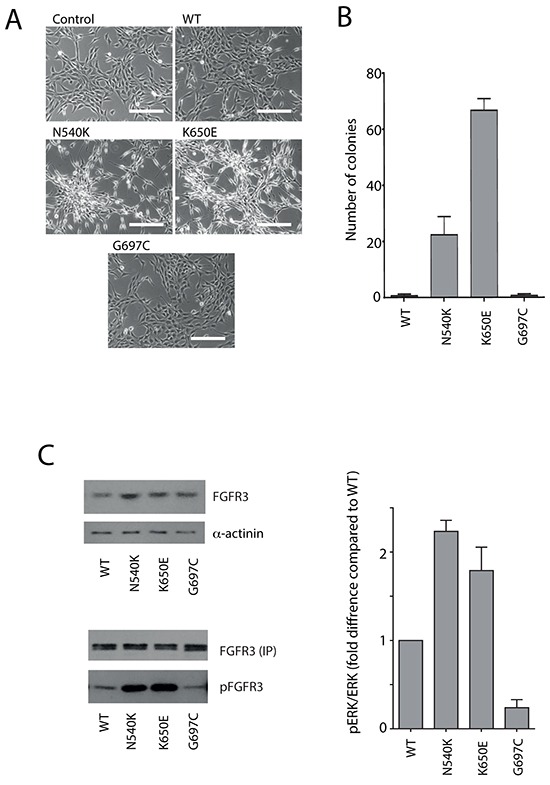
Analysis of G697C substitution in a cellular setting Control NIH3T3 cells and NIH3T3 cells expressing FGFR3 WT, N540K, K650E or G697C variants were analyzed for: transformed phenotype (scale bar represents 200 μm) **A.** anchorage independent growth **B.** FGFR3 expression level (**C.** left top) and ERK phosphorylation status shown as pERK/ERK ratio (**C.** right). Following FGFR3 immunoprecipation (with normalization for equal amounts) (FGFR3 IP), the phosphorylation status (pFGFR3) was subsequently assessed using anti-pY antibodies (**C.** left bottom). For the representative blots in panel **C**, lysates were prepared from cells subjected to serum withdrawal for 2 hrs in two separate experiments. See also Supplementary Figure S6.

It could be expected that some mutations that map to the KD do not affect kinase activity directly as measured under conditions *in vitro*. In particular, the hotspot G697C mutation which does not have an effect in such assays (Figure 2A and 2B, bottom panel) could impact on FGFR3 function in a way that can only be detected in a cellular setting. To test this possibility, a stably transfected FGFR3 G697C NIH3T3 cell line was compared with cell lines expressing two other hotspot variants, K650E and N540K, and the WT FGFR3 cell line (Figure 3). In contrast to cell lines expressing FGFR3 K650E and N540K mutations, FGFR3 G697C NIH3T3 cell line did not show a transformed phenotype (Figure 3A) or anchorage independent growth (Figure 3B); there was no detectable increase in downstream phosphorylation events and levels of phospho-FGFR were comparable to the WT (Figure 3C).

**Figure 4:**
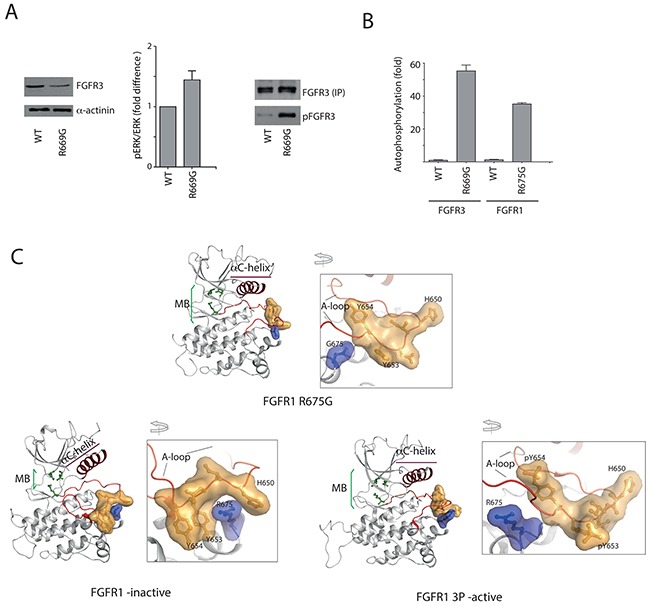
Impact of R669G substitution on function of FGFR3 in a cellular setting and on FGFR KD structure **A.** NIH3T3 cells expressing FGFR3 WT or R669G variant were analyzed for FGFR3 expression level (left) and ERK phosphorylation status shown as pERK/ERK ratio (middle); following FGFR3 immunoprecipation (with normalization for equal amounts) (FGFR3 IP), the phosphorylation status (pFGFR3) was subsequently assessed using anti-pY antibodies (right). See also Supplementary Figure S6. **B.** Auto-phosphorylation of purified kinase domain proteins: FGFR3 WT, FGFR3 R669G, FGFR1 WT and FGFR1 with the corresponding substitution R675G. Activity of each variant is expressed as a fold change compared to the FGFR3 WT (WT=1). **C.** FGFR1 R675G (PDB: 5FLF) (top), Apo FGFR1 (PDB: 4UWY) (bottom left) and FGFR1 3P-active (PDB: 3GQI) (bottom right) are represented as a cartoon diagram. The A-loop and αC helix are coloured in chocolate. The molecular break (MB) residues N546, E562 and K638 are represented as ball-and-stick model (green). The residues at the activation loop H650, I651, D652, Y653, Y654 and R/G 675 are shown as ball-and-stick-model along with a surface around them. The R/G residues are coloured in blue while the nearby residues surrounding R675 are coloured in orange. The phospho-tyrosine (pY) residues in FGFR1 3P-active form are represented with their van der Waals surface as spheres.

Analysis of the FGFR3 R669G NIH3T3 cell line has shown that despite low expression levels, downstream signaling appeared to be enhanced as well as FGFR3 phosphorylation (Figure 4A).

Comparison of our experimental data (Figure 2) with the assessments obtained using bioinformatics tools (Supplementary Table S1B and S1C) suggests that considering multiple methods together can provide insight into the influences of many panel mutations. Condel predicts three pathogenic mutations (E466K, D617G and G637W) that either reduce protein production or completely inactivate the kinase. Stabilization of active kinase is indicated by FOLDX with good specificity, but low sensitivity. However, most highly activating or moderately activating mutations have supporting evidence from either FOLDX or clustering (Supplementary Table S1B, Supplementary Figure S2). In particular, although not a hotspot, R669 is within an identified cluster of observed A-loop cancer mutations.

### Activation mechanism of FGFR3 R669G mutation

The residue corresponding to R669 in FGFR3 is conserved and also mutated in all other FGFRs in cancer as well as in FGFR2 in bone dysplasia (Supplementary Table S1). To assess the mechanism that underpins activation, we first analyzed the R to G replacement in the context of FGFR1 KD that is more amenable to crystallography compared to FGFR3 KD. As shown in Figure 4B, R675G FGFR1 KD variant also has higher activity compared to the WT. X-ray data collection and refinement statistics for the R675G FGFR1 KD 3D structure are summarized in Supplementary Table S2.

Comparison of this new FGFR1 R675G KD structure (Figure 4C, top) with inactive (apo) FGFR1 KD (PDB: 4UWY) and active, FGFR1-3P (pdb 3GQI) (Figure 4C, bottom) structures shows that FGFR1 R675G KD differs from the inactive form and adopts a fold with great similarity to the active KD. Notably, in the inactive FGFR1 KD there is direct hydrogen bonding between R675 and H650 from the A-loop; in addition, R675 is found to share a tight van der Waals interaction with active site tyrosine Y653 and H650 (Figure 4C, bottom left inset). These interactions are disturbed and broken in R675G mutant (Figure 4C, top inset), thus favoring the A-loop to adopt an open conformation as found in the active FGFR1-3P (Figure 4C, bottom right inset). In turn, this open conformation of the A-loop leads to several rearrangements of various control elements in the KD – including ordering of spine residues, dissociation in the molecular brake interacting residues, repositioning of the αC-helix and movement of the N-lobe toward the C-lobe.

To gain further insight into the activation mechanism of the R669G mutation in FGFR3, we performed NMR studies in which we compared the backbone amide chemical shift perturbations (CSPs) associated with the R669G mutation with those of the WT FGFR3 KD. Our results show that in addition to localized differences around the mutation, there are other differences in distant elements, consistent with a broader conformational change (Supplementary Figure S3). Of particular note are the significant CSPs in the αC-β4 loop and at the C-terminal end of the αC-helix, which likely report on the release of the molecular brake by the R669G mutation. The CSPs in helix αG and in the αF-αG linker also suggest altered interactions with the αEF helix and the activation loop, consistent with promotion of an active conformation.

Previous structural studies of FGFR2 KD highlighted a long-range allosteric communication linking the kinase hinge, the αC-helix and the A-loop [41]. It was also illustrated that some A-loop mutations (such as FGFR3 K650E), by forcing an active conformation of the loop, can change *via* this allosteric network the position of the αC-helix and also dissociate the molecular brake [23, 41]. We suggest a similar allosteric mechanism for FGFR1 R675G and corresponding FGFR3 R669G mutation that is in this case triggered by the loss of inhibitory interactions in the vicinity of the A-loop that involve the R675/669 residue.

### Structural insights into drug binding

**Figure 5:**
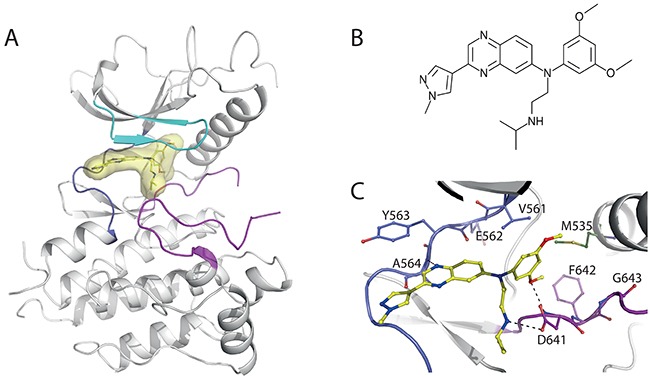
Structural insights into JNJ42756493 binding to FGFR1 KD **A.** Cartoon representation of JNJ42756493 (in yellow) bound FGFR1 KD. The A-loop is colored in purple, P-loop in cyan and the hinge region in dark blue. A van der Waals surface area is shown around the drug JNJ42756493. **B.** Chemical structure representation of JNJ42756493. **C.** A close-up view of JNJ42756493 (in yellow) and surrounding residues shown as ball-and-stick model.

Several recent structural studies revealed binding pockets of some selective (BGJ-398 and AZD4547) and non-selective (TKI258 and AP24534) FGFR inhibitors in complexes with FGFR1 KD [37, 42, 43]. For the FGFR-selective inhibitor JNJ42756493 there is much less reported information despite its promise for clinical use [44]. To help rationalize functional differences between these compounds we generated the structure of FGFR1 in complex with JNJ42756493 by soaking the compound into preformed crystals of FGFR1 KD in which there are two molecules of FGFR1 in the crystallographic asymmetric unit. The two monomers are highly similar, exhibiting rmsd values of 0.39 Å over 280 Å and 0.09 Å over 39 Å within 6 Å of the JNJ42756493 binding site. Further discussion will therefore refer to the structure of monomer A. The overall structure of FGFR1 KD bound to JNJ42756493 is shown in Figure 5A.

JNJ42756493 occupies the ATP-binding cleft of FGFR1 largely as expected on the basis of previous complexes between FGFR1 and other type-I inhibitors (e. g. BJG-398, AZD4547, PD173074 and TKI258) and where the activation loop clearly exhibits a DFG-in conformation. The quinoxaline core of JNJ42756493 is observed to form a single hydrogen bond to the hinge region via the main chain amide of A564 while the dimethoxyphenyl ring is orientated perpendicular to the quinoxaline core and occupies the hydrophobic pocket located behind the gatekeeper residue (V561). One of the methoxy oxygen atoms is involved in a hydrogen bond with the backbone nitrogen atom of the DFG aspartate (D641). The methyl pyrazole solubilizing group extends away from the hinge region towards the solvent channel and does not make any specific interactions with the protein. A structural comparison of various drug compounds (JNJ42756493, BGJ-398, AZD4547, TKI258 and AP24534; Supplementary Figure S4) bound to FGFR1 KD clearly indicates that a unique feature of JNJ42756493 is the amide side chain which extends into the region of the binding site normally occupied by the a-phosphate of ATP where it forms a hydrogen bond to the side chain of D641. In addition the terminal isopropyl group of this side chain also makes good van der Waals interactions with the protein in a shallow pocket formed by the side chains of N628, L630, A640 and D641 that has previously been referred to as the “pit” region [45]. Interestingly this indentation in FGFR1 has previously been found to be occupied by a methyl isoxazole moiety in a series of compounds containing a pyrazole core (PDB numbers: 4F64, 4F65, 4NK9, 4NKA and 4NKS). The side chain modification to JNJ42756493 therefore likely makes a significant contribution to its overall binding strength and specificity. Considering that JNJ42756493, BGJ-398, AZD4547, TKI258 and AP24534 are all in clinical trials, structural comparison of their binding to FGFR KD (Supplementary Figure S4) will contribute to understanding their clinical differences.

### Changes in drug efficacy due to activating mutations

It is well established that some acquired mutations in protein kinases greatly reduce drug binding; the best-illustrated examples are gatekeeper mutations also described in FGFR3 (V555M) [22, 37]. The question of how primary mutations in FGFR KDs, in particular activating mutations, affect drug efficacy has not been addressed directly although studies of FGFR2 resistance mutations to TKI258 using BaF3 cells suggested this possibility [46]. However, with a number of FGFR inhibitors now in clinical trials it is important to establish accurately their comparative efficacies towards different FGFR variants.

**Figure 6:**
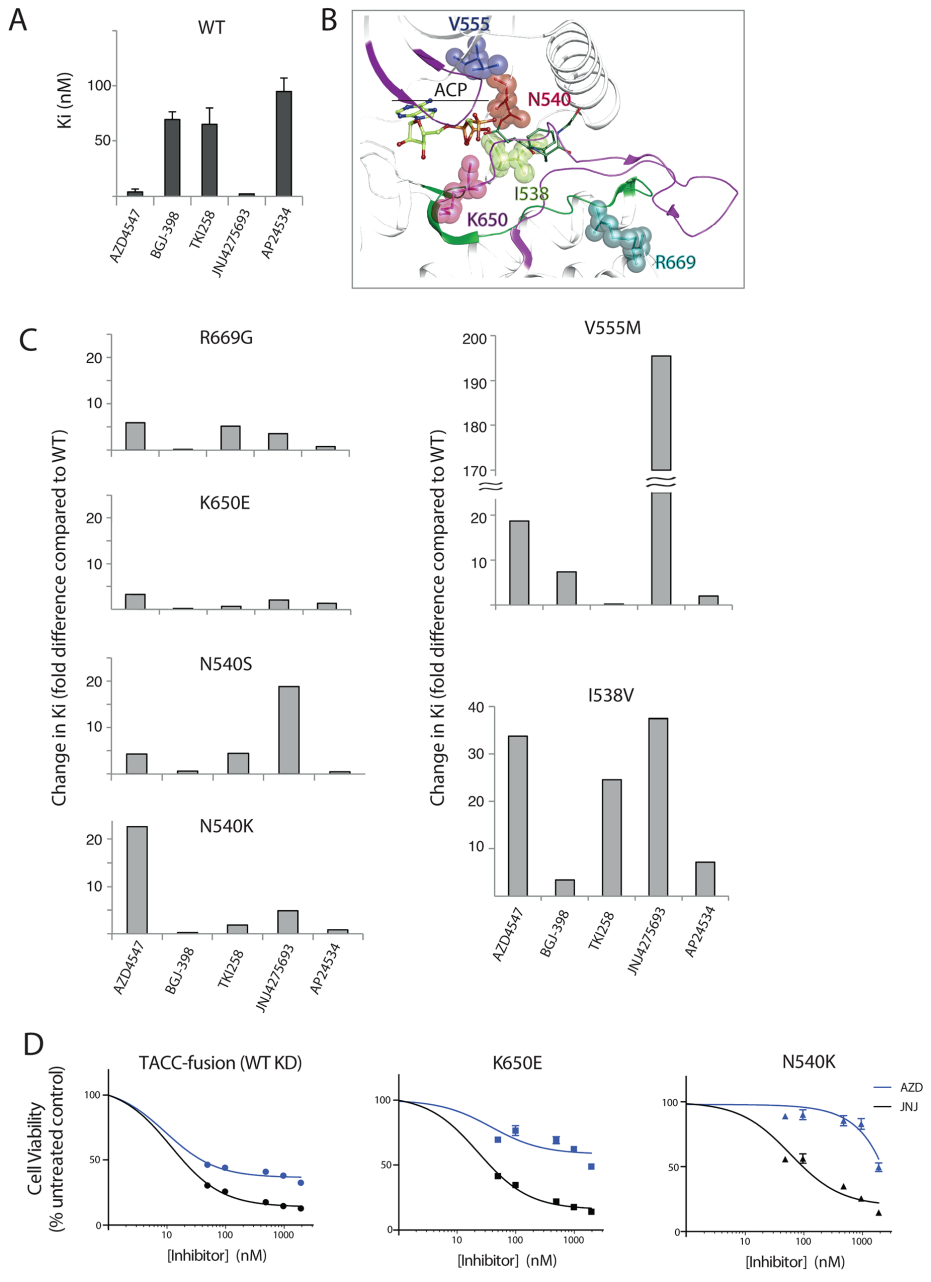
Inhibitor efficacy for a subset of FGFR3 variants measured *in vitro* and in cells **A.** Efficacy of indicated inhibitors on substrate phosphorylation by FGFR3 WT expressed as Ki values. **B.** A close-up view of FGFR1 KD structure in the complexes with an ATP-analogue, ACP (PDB: 3GQI). Residues corresponding to mutations in FGFR3 are highlighted and labeled using FGFR3 numbering. **C.** The effect of indicated substitutions on substrate phosphorylation by FGFR3 variants shown as difference in Ki compared to the wild type (WT=1). See Supplementary Table S3 for absolute values. **D.** Inhibitor efficacy in NIH3T3 stable cell lines. Efficacy of AZD4547 (blue) and JNJ42756493 (black) towards: FGFR3 with the WT KD (FGFR3TACC3-fusion) (left) and N540K (middle) or K650E FGFR3 (right) variants in NIH3T3 cells. Inhibitor concentrations used were 0, 50, 100, 500, 1000 and 2000 nM.

We performed measurements of Ki for AZD4547, BGJ-398, TKI258, JNJ42756493 and AP24534 using purified FGFR3 KD WT and variants R669G, K650E, N540S, N540K, V555M and I538V (Figure 6, Supplementary Table S3). Ki values for the WT FGFR3 KD show the first direct comparison of these compounds (Figure 6A); several previous studies focused on a single drug [18, 19, 47]. JNJ42756493 is the most effective (Ki about 2 nM), closely followed by AZD4547 (Ki about 4.5 nM) while another FGFR-specific inhibitor BGJ-398 has a Ki value similar to pan-kinase inhibitors TKI258 and AP24534 (within the range 65-95 nM) (Figure 6A, Supplementary Table S3). Mutations selected for this study include the gatekeeper (V555M) and several other activating mutations that are in the vicinity of the ATP binding pocket (N540S, N540K and I538V); R669G and K650E mutations are further away – however, they could have an allosteric effect (Figure 6B).

The impact of each mutation on drug binding is expressed as a fold-difference in Ki compared to the FGFR3 KD WT (Figure 6C). Highly activating R669G and, in particular, hotspot mutation K650E had moderate effects on the efficacy of all inhibitors; all changes for K650E were within 4–fold (Figure 6C, Supplementary Table S3). In contrast, mutations at the hotspot position N540 had a more pronounced effect (up to 23-fold) and most affected the compounds with low Ki values, with the N540K substitution affecting AZD4547 more and N540S affecting JNJ42756493 more. As expected, the gatekeeper V555M mutation, conferring resistance to AZ12908010 (similar to AZD4547), had an impact on the efficacy of AZD4547 (about 20-fold). The change in efficacy of JNJ42756493 due to V555M mutation was even greater (about 200-fold) representing the biggest change observed in this panel. Interestingly, as we reported previously [37], the affinity of TKI258 for V555M was higher compared to the WT. Residue I538 is in contact with N540 in the 3D structure [41] and the mutation I538V had substantial effect (up to 40-fold) on all inhibitors.

We further compared the effect of the two most potent FGFR-specific inhibitors AZD4547 and JNJ42756493 on hotspot mutations K650E and N540K in NIH3T3 cell lines. As previously reported [37] and shown in Supplementary Figure S5 NIH3T3 cells stably transfected with the “empty” expression vector are only marginally affected by FGFR inhibitors. An NIH3T3 cell line expressing FGFR3TACC3-fusion protein was used for comparison with the K650E and N540K NIH3T3 cell lines because the FGFR3 KD in this fusion has the WT sequence and, furthermore, this cell line has similarly transformed phenotype (Figure 6D). For the WT KD, AZD4547 and JNJ42756493 cellular IC_50_s reflect the *in vitro* differences of about 2-3 fold (Figure 6D, left and Supplementary Table S3). The K650E and N540K variants reduce efficacy of both compounds with the larger impact by the N540K mutation also showing a considerable difference between AZD4547 and JNJ42756493 for this mutant (Figure 6D, middle and right).

Some of the differences between the effects of tested inhibitors on activating FGFR variants (Figure 6) are consistent with observations from structural studies. Based on the crystal structure of FGFR1 KD V561M, the interactions of the inhibitors within the ATP-binding pocket, and their chemical structures (Supplementary Figure S1), it is expected that the efficacy of the JNJ42756493 towards FGFR3 V555M would be particularly affected. Contributing factors include the higher rigidity of this compound, compared with AZD4547 and BGJ-398, and the nature of occupancy of the ATP-binding pocket resulting in higher affinity (Figure 5, Supplementary Figure S1). In general, 3D structures alone are not sufficient to fully understand the mode and strength of drug binding [48] highlighting the importance of direct measurements performed here.

## DISCUSSION

Many clinically relevant cancer mutations were identified based on recurrence rates. However, this strategy cannot be readily applied to alterations that occur in a minority of tumors or to situations where different, rare mutations create the same neoplastic phenotype *via* different mechanisms. In fact, some of these mutations are of functional significance and likely constitute drivers, therapeutic targets or mechanisms of therapy resistance [49]. Here we have applied a direct comparative analysis of FGFR KD variants that represent the diversity of all mutations found in cancer and characterized their impact on kinase activity; for a subset of activating mutations we have also established their responsiveness to several drugs of particular clinical interest.

Our data reinforce the importance of frequently observed mutations at positions corresponding to FGFR3 N540 and K650 that occur in different FGFRs and in a range of cancer types as well as in bone dysplasia (Figures 1–3, Supplementary Table S1) [7, 9, 24]. In particular, replacements N to K and K to E at the respective positions are highly activating *in vitro* and transforming in NIH3T3 cells (Figures 2 and 3). In contrast, we have not been able to provide supporting evidence for an activating nature of FGFR3 mutation G697C (Figure 2 and 3). A relatively high number of observations of this mutation highlight FGFR3 G697C as another hotspot mutation (Figure 1). However, all observations of FGFR3 G697C were reported in a single study of oral squamous cell carcinomas (OSCC) [50]. It is possible that the activating potential of this mutation could be context dependent and involve interaction with another component(s) present in this type of epithelial cells. Interestingly, another study examining a comparable number of OSCC cases from a different population failed to detect FGFR3 G697C, arguing that this mutation is unlikely to be common even in this cancer type [51]. It is also conceivable that the occurrence of this mutation in a specific population could be unrelated to FGFR activation and involvement in OSCC. Further studies, focusing on FGFR3 G697C in epithelial cells rather than on comparative analysis shown here, would be needed to confirm this possibility.

A number of non-hotspot mutations have an impact on kinase activity, mainly resulting in moderate activation (Figure 2). At least some of these mutations are likely to have clinical relevance and they include a gatekeeper mutation (FGFR3 V555M) and a mutation (corresponding to FGFR3 I538V) also identified in a developmental disorder (hypochondroplasia) (Supplementary Table S1). The importance of mutations resulting in a lower degree of activation has been illustrated in developmental disorders, linking these mutations to less severe syndromes [52]; it is also possible that they could contribute to generation or progression of tumors or may be selected for after the selective pressure of anti-FGFR treatments. Moreover, we have established that an infrequent cancer mutation corresponding to R669G in FGFR3, also found in bone dysplasia, greatly enhances kinase activity of FGFR (Figures 1 and 2). One observation that could be related to its low reported frequency in cancer is the finding of low expression levels of this mutation in transfected mammalian cells (compared to FGFR3 N540K, K650E or WT) (Figure 4) that might compromise its impact or could require additional changes in cancer cells to increase its stability or expression. Nevertheless, structural studies of this mutation in the context of FGFR1 KD (R675G) are fully consistent with its activating potential. Furthermore, we here reveal a new feature of FGFR KD that stabilizes the inactive conformation which, when disrupted by mutations, can result in activation – namely, a set of interactions in the vicinity of the A-loop that involve R669/675 (Figure 4). Our new structural insights also consolidate observations resulting from structural studies of several other activating variants including FGFR2 N549H/T mutations in the molecular brake, FGFR2 K659N and FGFR3 K650E in the A-loop and FGFR1 V651M gatekeeper mutation affecting the regulatory spine [23, 37, 41]. Although R669/675 and these other mutations affect different regions in FGFR KD, the local structural changes trigger alterations in FGFR KD allosteric networks resulting in a shift towards conformations resembling an active form of phosphorylated kinase.

Combined with several recent structural studies of inhibitory compounds bound to FGFR KDs [37, 42, 43], our crystal structure of the JNJ42756493/FGFR1 KD complex provides a detailed picture of common features and key differences in the extent and nature of their binding interactions (Figure 5, Supplementary Figure S4). While these structures provide an important basis for understanding and improving the function of FGFR inhibitors, there are also limitations, in particular understanding the consequence of specific FGFR mutations requires direct experimental assessment. Indeed, our direct measurements of Ki values *in vitro* and evaluation of effects in cells show a complex picture where different mutations, including replacements of the same residue, can have very profound effects on the efficacy of some inhibitors but not on others (Figures 6C and 6D). In the case of FGFR3 acquired gatekeeper mutation (V555M), this is illustrated by about 200-fold reduction in efficacy for JNJ42756493 and an increase in efficacy for TKI258 (Figure 6C). Considering a good correlation between *in vitro* and *in vivo* effects of FGFR inhibitors in our previous study [37], these data are likely to be relevant for a cellular setting. Consistent with the *in vitro* data, we have observed loss of efficacy for PD173074 with the effect of TKI258 retained following introduction of FGFR3 V555M mutation in a cell line originally responding to both drugs [37]. Because JNJ42756493 is also effective towards FGFR4 [20], these findings could be important when considering the use of JNJ42756493 as a first-line treatment; primary, activating mutations corresponding to gatekeeper replacements have been reported in rhabdomyosarcoma (FGFR4 V550L and V550E) and in breast cancer (FGFR4 V550M) [16, 38]. Another example that could inform the use of FGFR-selective inhibitors in the clinic is the effect of the hotspot N540K mutation on JNJ42756493 and AZD4547 that shows a larger impact on AZD4547 both *in vitro* and in cells (Figures 6C and 6D). Differential responses (response to TKI258 and AP24534 compared with reduced sensitivity to PD173074) due to the mutation corresponding to N540K, introduced in FGFR2 in the context of the JHUEM-2 cancer cell line, have been previously observed [46].

For aberrations where the FGFR KD retains the WT sequence (such as overexpression and a number of fusion proteins) the efficacy of both JNJ42756493 and AZD4547 could be expected to be high, as we illustrate here using the WT protein *in vitro* and FGFR3TACC3-fusion in cells (Figures 6A and 6D). More broadly, previous assessments of efficacy of several FGFR-specific inhibitors in separate studies have shown a good response in cancers with gene amplification of the FGFR WT or fusion proteins; these studies were based on available panels of cancer cell lines and tumor xenografts [22, 42, 53–55]. However, there are only a few cancer cell lines harboring point mutations in the FGFR KD with just two mutations being represented, FGFR3 K650E and FGFR2 N550K. Due to various changes that co-occur with FGFR mutations in each of these cell lines, it was difficult to assess and compare their responses and obtain conclusive data. Therefore, our studies of drug efficacy performed *in vitro* and in stable cell lines, complement and expand these previous assessments and reveal differential, drug-specific impacts of different FGFR KD mutations.

## MATERIALS AND METHODS

### Generation of FGFR variants, protein purification and kinase assay

Proteins were expressed and purified as outlined in Bunney et al. [37]. Protein purity, integrity and phosphorylation status was assessed by native mass spectrometry and SDS-PAGE.

Auto-phosphorylation and substrate phosphorylation assays *in vitro* were performed using the ADP-Glo (Promega) methodology. Data for Ki values were analyzed using Graphpad Prism software where each data point was repeated in duplicate and the standard error of the mean presented as outlined in Bunney *et al.* [37]. A detailed description of the assay procedures can be found in the Supplementary Materials.

### Generation and analysis of stable NIH3T3 cell lines

Cell culture, preparation of stable cell lines, protein analysis (including Western blotting and immunoprecipitation) and anchorage independent growth assays were essentially as previously described [21, 37, 56] and are detailed in the Supplementary Materials. Quantitation of Western blots was performed by Image Studio™ Lite and the standard deviation was calculated from two independent data sets. Residues of the full-length FGFR3 IIIb were labeled consistently with the KD numbering (based on IIIc).

For the cell viability assay in the presence of FGFR inhibitors, NIH3T3 cells transfected with the stated FGFR3 variants were plated in 96-well plates, 1000 cells per well. Twenty-four hours later the cells were treated with various concentrations of kinase inhibitor. Forty-eight hours later, fresh inhibitor was added and the cells incubated for 72 hours. Cell viability was measured through the addition of CellTiter-Glo (Promega) and luminenscence measured with a standard luminometer plate reader. Data were normalised and fitted to a log(inhibitor) vs. response curve (three parameters) with GraphPad Prism.

### Crystallization, crystallography and NMR spectroscopy

FGFR1 crystals were grown by both, sitting and hanging drop methods and diffraction data collected at the Diamond Light source. Please refer to Supplementary Table S2 for X-ray data processing statistics. Phasing, refinement and structure validation are described in the Supplementary Materials.

The refined and validated structures of FGFR1^R675G^ and FGFR1 bound to JNJ42756493 have been submitted to Protein Data Bank (PDB) and their PDB ID codes are 5FLF and 5EW8 respectively.

For NMR spectroscopy, FGFR3 KD constructs, either the WT sequence or harboring R669G mutation, were uniformly labeled with ^15^N and purified as described in Bunney *et al.* [37]. ^1^H-^15^N TROSY-HSQC NMR spectra were acquired at 25°C.

### Computational methods

We obtained pan-cancer mutation frequencies for each FGFR3c amino acid by collating somatic missense mutations from COSMIC v71 and other cancer genome sources, alongside germ line Skeletal Dysplasia mutations from UniProt. Mutants were assessed for predicted structural effects, pathogenicity and protein stability. For full computational methods see Supplementary Materials.

## SUPPLEMENTARY FIGURES AND TABLES












