# Supplementary material for: Landscape of activating cancer mutations in FGFR kinases and their differential responses to inhibitors in clinical use
Source: Oncotarget. 2016 Mar 16;7(17):24252–68. doi: 10.18632/oncotarget.8132 (PMC5029699; doi:10.18632/oncotarget.8132)
Supplement: Supplementary file 1 [file oncotarget-07-24252-s001.pdf]

SUPPLEMENTARY MATERIALS & METHODS

Cloning & site-directed mutagenesis

As described in [1]. Point mutations were introduced using site-directed mutagenesis with PfuUltra™ II Fusion HS DNA polymerase (Agilent Technologies) following the manufacturer's instructions.

FGFR3 kinase domain protein expression & purification

As described in [1].

Production of phosphorylated FGFR3 kinase domain proteins

Recombinant unphosphorylated FGFR3 kinase domain proteins at 2-10 mg/mL were incubated with 25 mM $MgCl_2$ and 10 mM ATP for 45 minutes at 22°C in order for autophosphorylation to occur. The kinase reaction was stopped with 50 mM EDTA and desalted on a HiLoad 16/60 Superdex 75 column (GE Healthcare) using an Akta Purifier (GE Healthcare) with Desalting Buffer (25 mM Tris.Cl pH 8.0, 10 mM NaCl, 1 mM TCEP). Peak fractions corresponding to FGFR3 were pooled and loaded on to a 1 mL Resource Q anion-exchange column (GE Healthcare) equilibrated in Q Buffer A (25 mM Tris.Cl pH 8.0, 1 mM TCEP). Differentially phosphorylated FGFR3 proteins were eluted with a very shallow gradient over 500 column volumes to 40% of Q Buffer B (25 mM Tris.Cl pH 8.0, 1 M NaCl, 1 mM TCEP). The resulting chromatogram showed five peaks, corresponding to the 0P, 1P, 2P, 3P and 4P phosphorylated forms of FGFR3. This was confirmed by native PAGE analysis of pooled fractions from each peak. The 4P peak fractions were pooled and concentrated to 1-2 mg/mL using Vivaspin concentration units (Vivaproducts). Protein was stored until required at -80°C after snap freezing in liquid nitrogen.

Crystallisation and crystallography

FGFR1^{R675G}

Crystals of FGFR1^{R675G} KD were grown by sitting drop vapour diffusion method on MRC 2-well plates. The protein (at 10 mg/ml concentration) was equilibrated at a ratio of 1:1 protein to mother liquor (20 % PEG 400, 0.75 – 2.0 M ammonium sulphate, 0.1 M magnesium chloride, 0.1 M Hepes pH 7.5) and incubated at 16 °C. The crystals grew in about 5 – 7 weeks. X-ray diffraction data were recorded on beamline I04 at Diamond Light Source. The data were processed, integrated and scaled to a high resolution of 2.58 Å using XDS [2] and aimless [3] suite respectively (see Supplementary Table S2) in primitive orthorhombic space group. Initial phases of FGFR1^{R675} KD were calculated using molecular replacement [4] method in Phaser software suite [5] after trimming of all the loop regions in apo FGFR1 KD (PDB: 4UWY [1]). There were a total of 5 molecules per asymmetric unit with a Matthews coefficient of 2.7 and solvent content of 54.6 %.

Model building and refinement were performed using Phenix suite [6]. A subset of 5 % of reflections were kept aside for Rfree calculation. The structure was refined to a final Rcryst/Rfree value of 0.1937/0.2545 and rmsd in bond lengths and angles were 0.003 Å and 0.752 Å respectively. There were a total of 94.5 and 5.5 % of residues in the favoured and additionally favoured regions of Ramachnadran plot. The refined structures were validated using validation tools in Phenix [6] and online server Molprobity [7].

FGFR1 complex with JNJ42756493

FGFR1 wild-type protein was produced as described in Norman, R. *et al* (2012) [8]. Protein was crystallised using the hanging-drop vapour diffusion method at 277K. Drops of equal volumes of protein and reservoir solutions were suspended over a reservoir of 18-20% (w/v) PEG8000, 200mM ammonium sulphate, 100mM PCTP pH 6.75 and 20% (v/v) ethylene glycol. For soaking experiments, a solution of 22%(w/v) PEG8000, 200mM ammonium sulphate, 100mM PCTP pH 6.75, 20% (v/v) ethylene glycol and 1mM AZ13793811 was used and soaks were left to incubate for 24 hours. All work was carried out at 277K. Crystals from soaking experiments could be flash frozen in a stream of nitrogen gas at 100K directly from the drop. Diffraction data were collected at 100 K at I04-1 beamline at the Diamond synchrotron (Oxford, UK) on a Pilatus 6M detector. The data was integrated using the program XDS [2] and scaled with Aimless [3] as implemented in autoPROC [9] to a final resolution of 1.69Å. The FGFR1-JNJ crystals belong to the space group C1 2 1 and contain two complexes per asymmetric unit. The structure was solved by molecular replacement using the program PHASER [5] and an in-house FGFR1 structure as a search model. Subsequent model building and refinement were conducted using COOT [10] and BUSTER [11]. Quality checks were carried out using validation tools in COOT and Molprobity [7], while the compound stereochemistry was checked against the Cambridge Structure Database (CSD) [12] using Mogul [13].

Supplementary Table S2 gives a summary of the key data collection and refinement statistics.

PyMOL software suite (www.pymol.org) was used to prepare all the figures.

In vitro kinase assays

Kinase assays were carried out *in vitro* using the ADP-Glo™ Kinase Assay (Promega). Kinase reactions were carried out in Kinase Reaction Buffer (50 mM HEPES.Cl pH 7.5, 20 mM NaCl, 20 mM $MgCl_2$, 100 µM Na_3VO_4 and 100 µM TCEP) in 15 µL triplicates. All reactions in which the final kinase concentration was below 0.5 µM also contained 2 mM $MnCl_2$ and 0.1 mg/mL BSA. A poly-Glu-Tyr ($PolyE_4Y_1$) peptide (Sigma) was used as a synthetic kinase substrate. All reaction components were serially diluted in Kinase Reaction Buffer. Reactions were started by the addition of either ATP or kinase, depending on the type of assay. After the required duration, kinase reactions were stopped by the addition of 15 µL ADP-Glo™ Reagent for 40 minutes, followed by the addition of 30 µL Kinase Detection Reagent (KDR) for 30-60 minutes (depending on the concentration of ATP in the kinase reactions). Luminescence was measured at 520 nm on a FLUOstar Optima microplate reader (BMG Labtech). Data were analysed using Prism software (GraphPad). For Ki determination assays the method outlined in [1] was followed.

Cell culture

NIH 3T3 cells were cultured at 37°C and 10% CO_2 in medium consisting of high-glucose Dulbecco's Modified Eagle's Medium (DMEM) (Sigma) supplemented with 10% Fetal Bovine Serum (FBS) (Biosera or Gibco) and 1% GlutaMAX™ (Life Technologies). Stable FGFR3b NIH 3T3 cell lines, generated by retroviral transduction, were cultured similarly with medium supplemented with hygromycin B (Invitrogen) at a maintenance dose of 100 µg/mL.

Phoenix™ cell lines were cultured at 37°C and 5% CO_2 in the same medium as above supplemented with hygromycin B at a maintenance dose of 300 µg/mL.

Production of stable NIH 3T3 cell lines

Full length FGFR3 constructs were generated in the pFB retroviral expression vector (Stratagene) modified to contain a hygromycin b resistance cassette [14]. Plasmids were prepared using an alkaline lysis large-scale plasmid preparation method. Two point five µg of purified plasmid was then mixed with TransIT-293 Transfection Reagent (Mirus) in the presence of serum-free medium for 20 minutes at room temperature. Complexes were transfected into Phoenix™ cell lines and maintained daily as described above. After 72 hours the retroviral supernatant was harvested and mixed with 8 µg/mL polybrene (Sigma). NIH 3T3 cells were incubated in the retroviral supernatant for 4 hours, and then maintained in medium until they were considered 'stable' (two passages post-transduction). Successfully transduced cells were propagated by selection with 200 µg/mL hygromycin B before freezing the stable cell lines. Upon re-seeding from stocks, cell lines were subjected to selection with medium containing 200 µg/mL hygromycin b for a further three passages before they were fully selected. Cells were then maintained in medium containing maintenance dose hygromycin b at 100 µg/mL.

Protein extractions & immunodetection

Cultured cells were starved for 2 hours at 37°C and 10% CO_2 in FBS-free medium and then lysed *in situ* in Lysis Buffer (20 mM Tris.Cl pH 7.5, 150 mM NaCl, 1 mM EDTA, 1 mM EGTA, 1% [v/v] Triton, 20 mM sodium pyrophosphate, 25 mM sodium fluoride, 1 mM β-glycerophosphate, 3 mM Na_3VO_4, 1 µg/ml leupeptin) containing cOmplete™ protease inhibitor cocktail (Roche). Lysates were clarified by centrifugation at 16,100 x *g* for 15 min at 4°C. Total protein concentration was determined using the Pierce™ BCA Protein Assay Kit (Thermo Scientific) and 15 µg protein was run on 10% SDS-PAGE gels (produced in-house). Proteins were transferred to PVDF or nitrocellulose membranes using the Trans-Blot SD Semi-Dry Transfer Cell (Bio-Rad) and blocked in 5% [w/v] NFDM or 3% [w/v] BSA respectively. Membranes were incubated with primary antibodies for 14 hours at 4°C. Primary antibodies used were anti-β-actin (1:2000 in 3% [w/v] NFDM) (Abcam), anti-FGFR3 clone B9 (1:1000 in 3% [w/v] NFDM) (Santa Cruz Biotechnology), anti-ERK (1:1000 in 3%[w/v] NFDM) (Millipore) and anti-phosphoERK (1:2000 in 3 % [w/v] BSA) (Cell Signaling Technology). These were all used with HRP-conjugated secondary antibodies and ECL Prime (GE Healthcare) and imaged using the Odyssey Fc System (Li-Cor Biosciences) or Super RX Fuji medical x-ray film (Kodak). For immmunoprecipitation, protein samples (normalized for FGFR3 amount using a separate aliquot) were incubated with antibodies to FGFR3 extracellular domain (F3922, Sigma) for 16 hours at 4°C, followed by the addition of protein A sepharose beads (Amersham Biosciences) for 4 hours. The beads were washed four times in PBS, resuspended in 2xSDS loading and subjected to SDSPAGE and Western blotting.

Anchorage independent growth assays

Stable NIH 3T3 wildtype and mutant cell lines were seeded in triplicate at 5 x 10^3 cells per well of a 6-well plates in medium containing 0.4% agarose (Gibco), on a base of medium containing 0.8% agarose. Cells were fed weekly with medium containing 0.4% agarose. After two

weeks, cells were stained for 24 hours with 0.3% [w/v] p-iodonitrotetrazolium violet (Sigma) and each well was imaged using a Hamamatsu ORCA-285 camera coupled to a Nikon SMZ1000 microscope. Thresholding parameters for area and perimeter of colonies with a mean diameter greater than 100 μm were set on ImageJ. Colonies were counted only if they satisfied both the area and perimeter criteria above the threshold values.

Computational methods

Sequences

Sequences were downloaded from UniProt in FASTA format for FGFR1 (P11362-1), FGFR2 IIIc (P21802-1), FGFR3 IIIc (P22607-1) & FGFR4 (P22455-1). Alignments used default settings of ClustalOmega [15] via Jalview 2.8.2 [16]. FGFR3 canonical amino acid numbering refers to FGFR3c (UniProt: P22607-1).

Structures & preparation

For structural studies we used "F3-Active", the active-like conformation of an FGFR3 K650E mutant (PDB ID: 4k33 [17]) and "F3-Apo" a model of FGFR3 inactive/*apo* kinase generated with Modeller [18] based on FGFR1 kinase-domain structure in an inactive/*apo* conformation (PDB ID: 4uwy [1]). Model quality was checked using MolProbity [7], ProSA-web [19], ModEval [20] and DOPE [21]. In addition, the model has a very high SSAP score [22] (>90) when compared to F3-Active, with a corresponding RMSD of 2.45Å.

For FOLDX analysis using F3-Active, three mutations found in PDB structure 4k33 were back-mutated, changing S482 to C, S582 to C and E650 to K to match the canonical FGFR3c sequence. UCSF Chimera "Dock Prep" tool [23] was used on both F3-Active and F3-Apo to remove ions, ligands and solvent and replace missing side-chains, add hydrogen atoms and AMBERff14SB [24]-calculated charges, followed by energy minimisation.

Mutations

The majority of somatic mis-sense mutations were obtained using COSMIC v71 (cancer.sanger.ac.uk) [25]. To ensure a more comprehensive assessment of rare mutations, we also included data from TCGA (TCGA Research Network: http://cancergenome.nih.gov/), ICGC (International Cancer Genome Consortium https://icgc.org/) and BioMuta [26]. Downloaded COSMIC mutations were reported across all cancer types for entries annotated as 'Substitution - Mis-sense' and having a specific amino acid mutation call. Simple Somatic Mutations were downloaded from ICGC (v17) and BioMuta (1st December 2014) for FGFRs1, 2, 3 & 4. TCGA data in MAF (Mutation Annotation File) format was obtained for 19 cancer types (ACC, BLCA, BRCA, CESC, COAD, COADREAD, GBM, GBMLGG, HNSC, KICH, KIRC, KIRP, LAML, LGG, LIHC, LUAD, LUSC, OV & PAAD) using Broad Institute GDAC Firehose [27] on 6th December 2014. Annotations of variants at the amino acid level were obtained for each cancer type using Ensembl Variant Effect Predictor (VEP) [28]. Care was taken to avoid duplicating mutation counts, for example, by not counting ICGC mutations that originated from TCGA twice. For FGFRs1-4 we combined all data sources, aggregating mutation counts both by the total number at each amino acid position and by the number of specific mutations found (i.e. K650E, K650M, etc).

Germline mutations were downloaded on 17th June 2015 from both UniProt Human Variants ('http://www.uniprot.org/docs/humsavar.txt) and Clinvar (http://www.ncbi.nlm.nih.gov/clinvar/). In addition, we referred to a review [29] to determine which amino acid positions on FGFR3 had reported Dysplasia mutations coincident with cancer mutations in either FGFR1, 2 or 3.

FOLDX stability calculations

We used FOLDX version 3b6 [30] to assess effects of mutations on protein stability of all possible single nucleotide variant, mis-sense mutations in FGFR3 kinase domain. The FOLDX "Position Scan" function was used in script mode for F3-Active and F3-Apo, using five runs per mutation and enabling the "RepairPDB" option. Multiple runs allow averaging of energy differences arising from side-chain rotamer conformations. For each mutation on each structure we obtained the difference in free energy of protein unfolding caused by the change to mutant amino acid in $kcalmol^{-1}$ as:

$$\Delta\Delta G = \Delta G_{mut} - \Delta G_{wt}$$

We compared differential effects of mutations between F3-Active and F3-Apo using:

$$\Delta\Delta\Delta G = \Delta\Delta G_{Active} - \Delta\Delta G_{Apo}$$

Here, negative values of $\Delta\Delta\Delta G$ indicate stabilisation of F3-Active with respect to F3-Apo, indicating a shift in equilibrium that favours activated kinase. We categorised $\Delta\Delta\Delta G$ for each mutant using percentiles calculated from the total distribution of values for all possible mutants (5%: Stabilising - very high, 10%: Stabilising- high, 20%: Stabilising - medium and 30%: Stabilising - low; destabilising mutants were categorised equivalently). The middle 40% of $\Delta\Delta\Delta G$ values were not annotated as either stabilising or destabilising.

Clustering of mutations on structure

In any given tumour sample most mutations are extremely rare, thus lack of reported mutations of specific residues may reflect insufficient coverage by

cancer genomics projects. In identifying potential novel driver mutations, residues that are part of a cluster of observed mutations on the protein structure are reasonable candidates. We used an in-house method "MutClust" to test whether the density of all observed mutations within a spherical volume centred on each amino acid in turn is greater than would be expected by chance, using the F3-Active structure.

Briefly, all known mutations across FGFRs1-4 were mapped to F3-Active. For each amino acid, we tallied mutation-harbouring residues within spheres of radii 4, 5, 6 & 7Å. The observed mutation counts in each sphere may reflect both random variation in underlying mutation rates and different local protein structure densities (for example, on average we would expect more neighbouring mutations of residues in the densely packed protein core compared to a loop region). To account for these, we developed a random model based on a permutation test. We generated random mutant structures by assigning each amino acid a discrete value as either mutated (1) or not (0) using a probability estimated from the data as:

$$P(r_i = 1) = \frac{1}{len(s)} \sum_{j=1}^{len(s)} s_j \mid r_i, s_j \in \{0,1\} \simeq 0.33$$

Where (for a given random mutant) r_i is the ith amino acid of randomised sequence r and s is the F3-Active sequence, where $s_j = 1$ if a mutation is observed at amino acid j in any of the databases outlined for any of FGFRs1-4, or 0 otherwise.

We used 1000 permutation tests for each sphere radius (4, 5, 6 & 7Å) allowing calculation of means & standard deviations of the number of neighbouring random mutations found for each amino acid at each sphere radius. Hotspots were defined as residues with more observed mutated neighbouring residues than by chance using 99% significance. Residues were assigned to one of two identified hotspot clusters by identifying the least overlapping sets of residues within the significant cluster spheres (see Supplementary Figure S2).

Pathogenicity predictions

Condel [31] (**con**sensus**del**eterious) assessed multiple methods for predicting deleteriousness of non-synonymous SNVs: SIFT [32], Polyphen2 [33], MutationAssessor [34] and FATHMM [35]. Condel v2 data (downloaded via FannsDB at http://bg.upf.edu/fannsdb/ on 28th Nov 2014) uses a consensus of MutationAssessor and FATHMM. Condel scores were converted to z–scores using their values over the F3-Active kinase-domain. We defined 'highly deleterious' mutations as those having Condel $z > Q_3$ (Q=Quartile).

F3-Active mutations occurring in cancer or germline were submitted to SAAPdb [36]. SAAP predicts structural effects (e.g. disruption of H-bonding, salt-bridges, clashes, burying charge) and reports highly conserved sites and known functional sites from UniProt (e.g. binding sites, active sites).

Mutations submitted for SAAPdb analysis were also submitted to SAAPpred [37], a machine learning method that classifies SAAP features, outputting a prediction of neutral (SNP) or pathogenic (PD) and a confidence score.

Technical implementation

We used SQL scripts to import, combine and filter data, using a custom-built database. Further processing of data for structural clustering and FOLDX calculations used R scripts, with final presentation using R [38] & Microsoft Excel.

REFERENCES

1. Bunney TD, Wan S, Thiyagarajan N, Sutto L, Williams SV, Ashford P, Koss H, Knowles MA, Gervasio FL, Coveney PV and Katan M. The Effect of Mutations on Drug Sensitivity and Kinase Activity of Fibroblast Growth Factor Receptors: A Combined Experimental and Theoretical Study. EBioMedicine. 2015; 2:194-204.
2. Kabsch W. Processing of X-ray snapshots from crystals in random orientations. Acta crystallographica Section D, Biological crystallography. 2014; 70:2204-2216.
3. Evans PR and Murshudov GN. How good are my data and what is the resolution? Acta crystallographica Section D, Biological crystallography. 2013; 69:1204-1214.
4. Rossmann MG. The molecular replacement method. Acta crystallographica Section A, Foundations of crystallography. 1990; 46:73-82.
5. McCoy AJ, Grosse-Kunstleve RW, Adams PD, Winn MD, Storoni LC and Read RJ. Phaser crystallographic software. Journal of applied crystallography. 2007; 40:658-674.
6. Adams PD, Afonine PV, Bunkoczi G, Chen VB, Davis IW, Echols N, Headd JJ, Hung LW, Kapral GJ, Grosse-Kunstleve RW, McCoy AJ, Moriarty NW, Oeffner R, Read RJ, Richardson DC, Richardson JS, et al. PHENIX: a comprehensive Python-based system for macromolecular structure solution. Acta crystallographica Section D, Biological crystallography. 2010; 66:213-221.
7. Chen VB, Arendall WB, 3rd, Headd JJ, Keedy DA, Immormino RM, Kapral GJ, Murray LW, Richardson JS and Richardson DC. MolProbity: all-atom structure validation for macromolecular crystallography. Acta crystallographica Section D, Biological crystallography. 2010; 66:12-21.

8. Norman RA, Schott AK, Andrews DM, Breed J, Foote KM, Garner AP, Ogg D, Orme JP, Pink JH, Roberts K, Rudge DA, Thomas AP and Leach AG. Protein-ligand crystal structures can guide the design of selective inhibitors of the FGFR tyrosine kinase. Journal of medicinal chemistry. 2012; 55:5003-5012.
9. Vonrhein C, Flensburg C, Keller P, Sharff A, Smart O, Paciorek W, Womack T and Bricogne G. Data processing and analysis with the autoPROC toolbox. Acta crystallographica Section D, Biological crystallography. 2011; 67:293-302.
10. Emsley P and Cowtan K. Coot: model-building tools for molecular graphics. Acta crystallographica Section D, Biological crystallography. 2004; 60:2126-2132.
11. Bricogne G, Blanc E, Brandl M, Flensburg C, Keller P, Paciorek P, Roversi P, Sharff A, Smart O, Vonrhein C and Womack T. (2010). BUSTER version 2.11.1. Global Phasing Ltd.).
12. Allen FH. The Cambridge Structural Database: a quarter of a million crystal structures and rising. Acta crystallographica Section B, Structural science. 2002; 58:380-388.
13. Bruno IJ, Cole JC, Kessler M, Luo J, Motherwell WD, Purkis LH, Smith BR, Taylor R, Cooper RI, Harris SE and Orpen AG. Retrieval of crystallographically-derived molecular geometry information. Journal of chemical information and computer sciences. 2004; 44:2133-2144.
14. Tomlinson DC, L'Hote CG, Kennedy W, Pitt E and Knowles MA. Alternative splicing of fibroblast growth factor receptor 3 produces a secreted isoform that inhibits fibroblast growth factor-induced proliferation and is repressed in urothelial carcinoma cell lines. Cancer research. 2005; 65:10441-10449.
15. Sievers F, Wilm A, Dineen D, Gibson TJ, Karplus K, Li W, Lopez R, McWilliam H, Remmert M, Soding J, Thompson JD and Higgins DG. Fast, scalable generation of high-quality protein multiple sequence alignments using Clustal Omega. Molecular systems biology. 2011; 7:539.
16. Waterhouse AM, Procter JB, Martin DM, Clamp M and Barton GJ. Jalview Version 2--a multiple sequence alignment editor and analysis workbench. Bioinformatics. 2009; 25:1189-1191.
17. Huang Z, Chen H, Blais S, Neubert TA, Li X and Mohammadi M. Structural mimicry of a-loop tyrosine phosphorylation by a pathogenic FGF receptor 3 mutation. Structure. 2013; 21:1889-1896.
18. Eswar N, Webb B, Marti-Renom MA, Madhusudhan MS, Eramian D, Shen MY, Pieper U and Sali A. Comparative protein structure modeling using MODELLER. Current protocols in protein science / editorial board, John E Coligan [et al]. 2007; Chapter 2:Unit 2 9.
19. Wiederstein M and Sippl MJ. ProSA-web: interactive web service for the recognition of errors in three-dimensional structures of proteins. Nucleic Acids Res. 2007; 35:W407-410.
20. Sali A and Blundell TL. Comparative protein modelling by satisfaction of spatial restraints. Journal of molecular biology. 1993; 234:779-815.
21. Shen MY and Sali A. Statistical potential for assessment and prediction of protein structures. Protein science : a publication of the Protein Society. 2006; 15:2507-2524.
22. Orengo CA and Taylor WR. SSAP: sequential structure alignment program for protein structure comparison. Methods in enzymology. 1996; 266:617-635.
23. Pettersen EF, Goddard TD, Huang CC, Couch GS, Greenblatt DM, Meng EC and Ferrin TE. UCSF chimera - A visualization system for exploratory research and analysis. J Comput Chem. 2004; 25:1605-1612.
24. Case DA, Darden T, Cheatham TE, Simmerling CL, Wang J, Duke RE, Luo R, Walker RC, Zhang W, Merz KM and al. e. AMBER 12. University of California, San Francisco. 2012; 1:3.
25. Forbes SA, Beare D, Gunasekaran P, Leung K, Bindal N, Boutselakis H, Ding M, Bamford S, Cole C, Ward S, Kok CY, Jia M, De T, Teague JW, Stratton MR, McDermott U, et al. COSMIC: exploring the world's knowledge of somatic mutations in human cancer. Nucleic Acids Res. 2015; 43:D805-811.
26. Wu TJ, Shamsaddini A, Pan Y, Smith K, Crichton DJ, Simonyan V and Mazumder R. A framework for organizing cancer-related variations from existing databases, publications and NGS data using a High-performance Integrated Virtual Environment (HIVE). Database : the journal of biological databases and curation. 2014; 2014:bau022.
27. (2015). Analysis-ready standardized TCGA data from Broad GDAC Firehose stddata 2014 12 06 run. In: Center BIoMaHBITGDA, ed.
28. McLaren W, Pritchard B, Rios D, Chen Y, Flicek P and Cunningham F. Deriving the consequences of genomic variants with the Ensembl API and SNP Effect Predictor. Bioinformatics. 2010; 26:2069-2070.
29. Wilkie AO. Bad bones, absent smell, selfish testes: the pleiotropic consequences of human FGF receptor mutations. Cytokine & growth factor reviews. 2005; 16:187-203.
30. Schymkowitz J, Borg J, Stricher F, Nys R, Rousseau F and Serrano L. The FoldX web server: an online force field. Nucleic Acids Res. 2005; 33:W382-388.
31. Gonzalez-Perez A and Lopez-Bigas N. Improving the assessment of the outcome of nonsynonymous SNVs with a consensus deleteriousness score, Condel. American journal of human genetics. 2011; 88:440-449.

32. Kumar P, Henikoff S and Ng PC. Predicting the effects of coding non-synonymous variants on protein function using the SIFT algorithm. Nature protocols. 2009; 4:1073-1081.
33. Adzhubei IA, Schmidt S, Peshkin L, Ramensky VE, Gerasimova A, Bork P, Kondrashov AS and Sunyaev SR. A method and server for predicting damaging missense mutations. Nature methods. 2010; 7:248-249.
34. Reva B, Antipin Y and Sander C. Predicting the functional impact of protein mutations: application to cancer genomics. Nucleic Acids Res. 2011; 39:e118.
35. Shihab HA, Gough J, Cooper DN, Stenson PD, Barker GL, Edwards KJ, Day IN and Gaunt TR. Predicting the functional, molecular, and phenotypic consequences of amino acid substitutions using hidden Markov models. Human mutation. 2013; 34:57-65.
36. Hurst JM, McMillan LE, Porter CT, Allen J, Fakorede A and Martin AC. The SAAPdb web resource: a large-scale structural analysis of mutant proteins. Human mutation. 2009; 30:616-624.
37. Al-Numair NS and Martin AC. The SAAP pipeline and database: tools to analyze the impact and predict the pathogenicity of mutations. BMC genomics. 2013; 14:S4.
38. Team RC. (2014). R: A language and environment for statistical computing. (Vienna, Austria: R Foundation for Statistical Computing).

SUPPLEMENTARY FIGURES AND TABLES

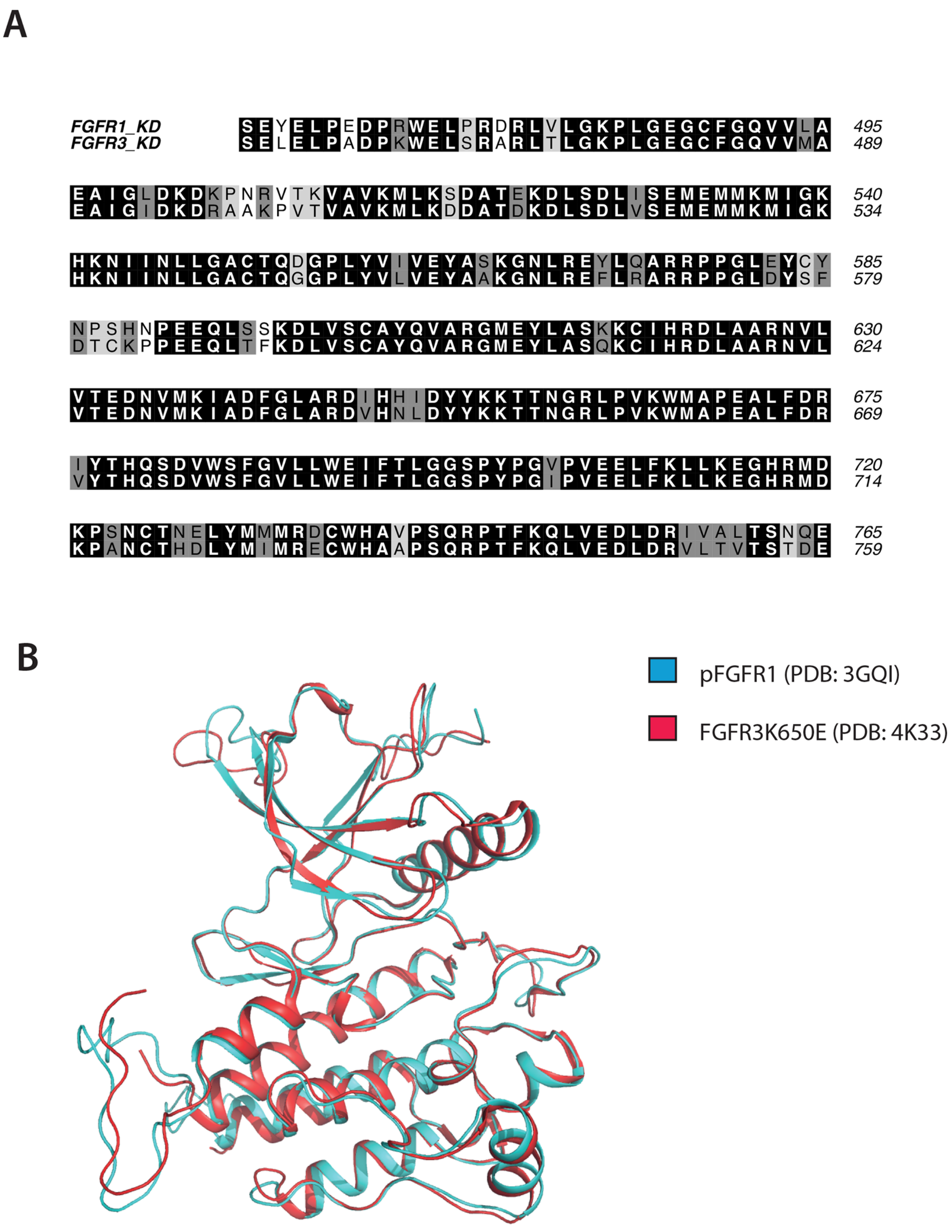

Supplementary Figure S1: Comparison of FGFR1 and FGFR3 kinase domains. A. Sequence alignment of kinase domains of FGFR1 and FGFR3. Identical residues are highlighted in black background while amino acids with similar properties (conservative substitutions) are highlighted in dark and light grey. Highlighted in dark grey are those amino acids that share similar charge. The proteins share 83% sequence identity and 92% sequence similarity. **B.** Cartoon representation and overlay of kinase domain of phosphorylated FGFR1 (PDB:3GQI) (blue) and activating mutant of FGFR3 (PDB: 4K33) (red). The secondary structure elements and overall structures are highly conserved between these two proteins.

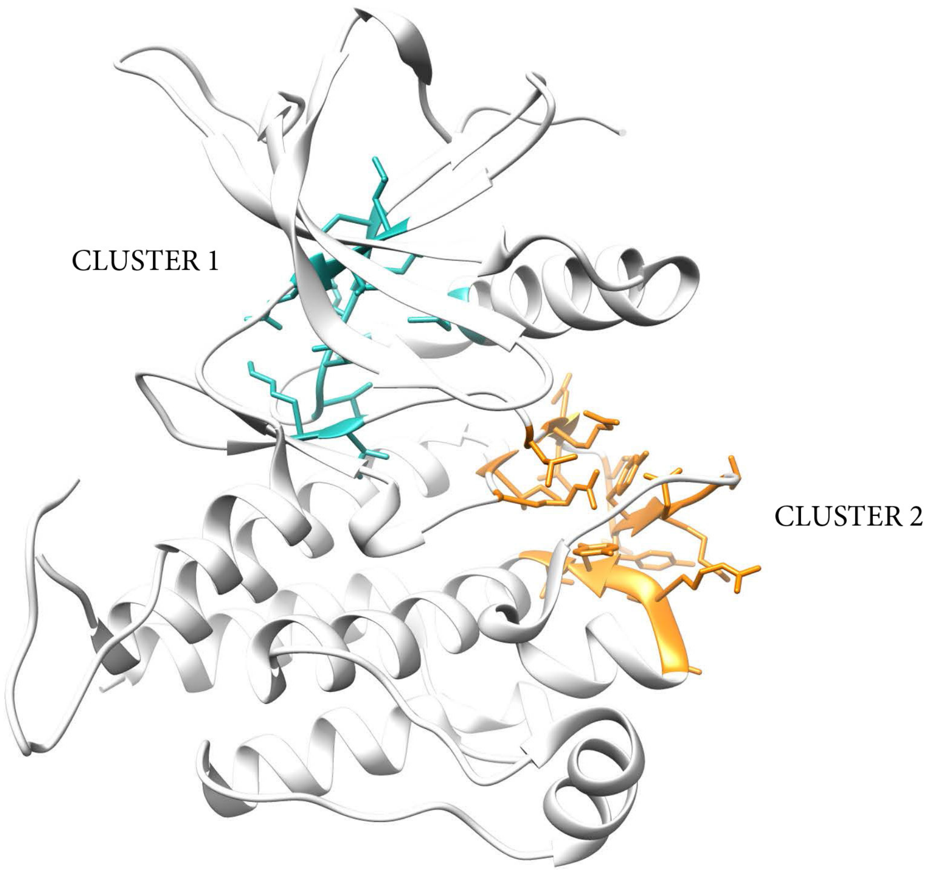

Supplementary Figure S2: "MutClust" mutation clusters on FGFR3. Two clusters of residues obtained from the MutClust algorithm indicate regions of high mutation density around the molecular brake (in cyan) and A-loop (in orange). Clusters are shown on PDB ID:4k33.

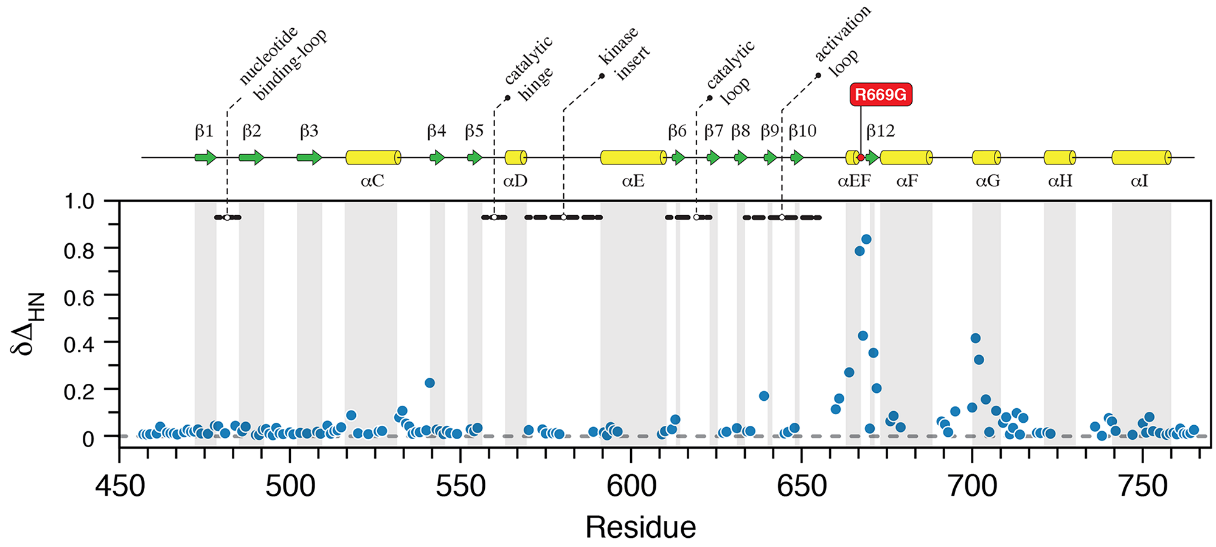

Supplementary Figure S3: HSQC Chemical shift perturbations in a comparison of the kinase domains of FGFR3^{WT} and FGFR3^{R669G}. Corresponding FGFR3 secondary structure elements and noteworthy functional elements are highlighted above the chemical shift plot as in main Figure 1D.

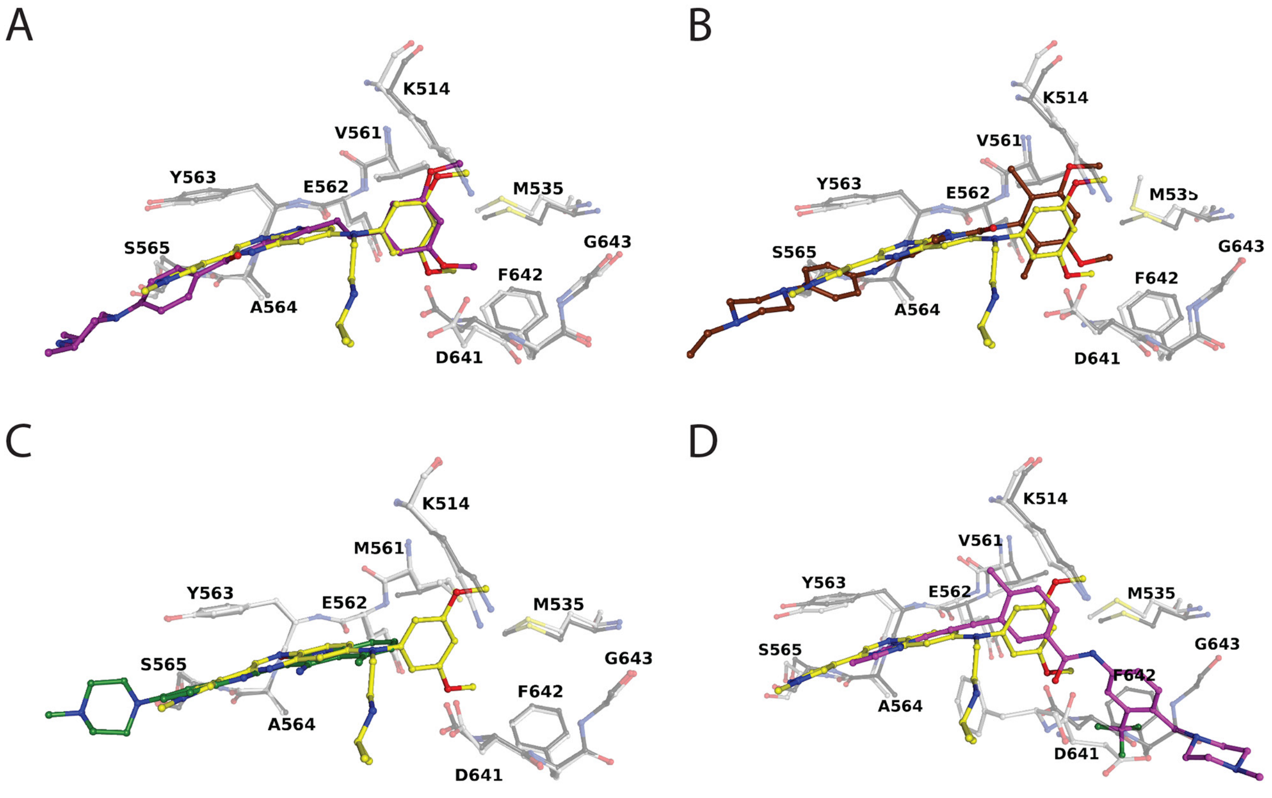

Supplementary Figure S4: Comparison of JNJ42756493 binding to FGFR1 with other inhibitors. A. Superposition of JNJ42756493 (in yellow) with AZD4547 (PDB: 4V05) (in purple). Nearby contact residues are shown as ball-and-stick model for JNJ42756493 (dark grey) and AZD4547 (light grey). **B.** Superposition of JNJ42756493 (in yellow) with BGJ-398 (PDB: 3TT0) (in chocolate). Nearby contact residues are shown as ball-and-stick model for JNJ42756493 (dark grey) and BGJ-398 (light grey). **C.** Superposition of JNJ42756493 (in yellow) with TKI258 (PDB: 5AM7) (in forest). Nearby contact residues are shown as ball-and-stick model for JNJ42756493 (dark grey) and TKI258 (light grey). **D.** Superposition of JNJ42756493 (in yellow) with AP24534 (PDB: 4V01) (in purple). Nearby contact residues are shown as ball-and-stick model for JNJ42756493 (dark grey) and AP24534 (light grey).

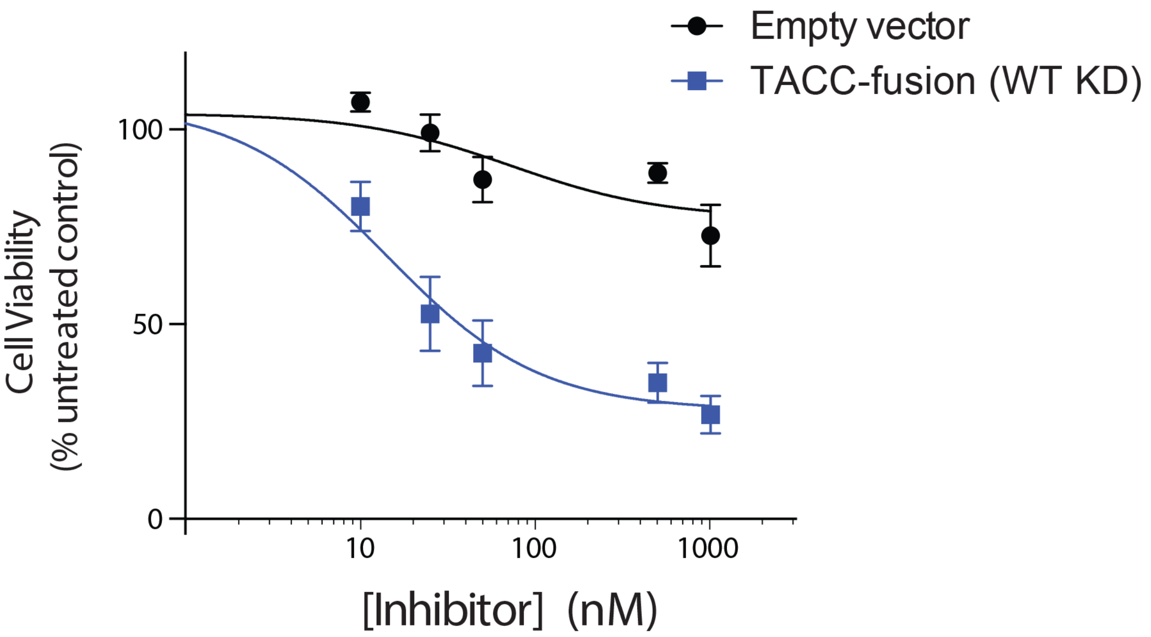

Supplementary Figure S5: Efficacy of AZD4547 in NIH3T3 stable cell lines. The efficacy towards NIH3T3 cell line expressing FGFR3 with the WT KD (FGFR3TACC3-fusion) was compared to NIH3T3 cell line stably transfected with the corresponding empty vector. The inhibitor concentrations used were 0, 10, 25, 50, 500 and 1000 nM.

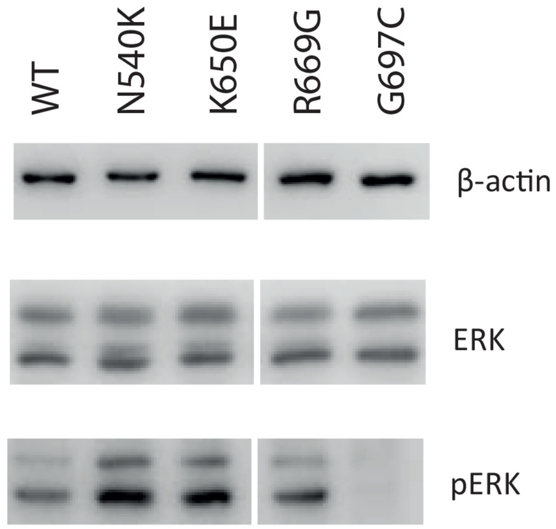

Supplementary Figure S6: Levels of ERK expression and phosphorylation in NIH3T3 stable cell lines. Cell lysates from NIH3T3 cells expressing FGFR3 WT, N540K, K650E, R669G and G697C variants were subjected to Western blotting using indicated antibodies. The white separation between the lanes shows that an intervening lane from the same blot was not included.

Supplementary Table S1a: Computational analysis of FGFR KD mutations.

See Supplementary File 1

Supplementary Table S1b: Summary of bioinformatics predictions of SNV effects.

See Supplementary File 2

Supplementary Table S1c: Summary of Overlap between Experimental and Computational Predictions.

See Supplementary File 3

Supplementary Table S2: X-ray data collection and refinement statistics.

See Supplementary File 4

Supplementary Table S3: Measurements of Ki for selected FGFR3 variants and inhibitors.

See Supplementary File 5
