# Supplementary material for: Landscape of activating cancer mutations in FGFR kinases and their differential responses to inhibitors in clinical use
Source: Oncotarget. 2016 Mar 16;7(17):24252–68. doi: 10.18632/oncotarget.8132 (PMC5029699; doi:10.18632/oncotarget.8132)
Supplement: Supplementary file 2 [file oncotarget-07-24252-s002.pdf]

Supplemental Table S1a: Computational Analysis of FGFR3 Kinase Domain Mutations.

ALIGNMENT	ANNOTATION	ALL MUTATIONS AT THIS AMINO ACID POSITION			SPECIFIC FGFR3 MUTATIONS			FOLDX STABILITY				CONDEL	SAAP			ALIGNMENT	
FGFR3_AA		FGFR1-4 total cancer mutations	Skeletal Dysplasia	3D cluster (MutClust)	WT	*MUT*	FGFR3 mutations	Apo *ΔΔG (kcal/mol)*	FGFR3 (4k33/WT) *ΔΔG (kcal/mol)*	*ΔΔΔG = ΔΔG(FGFR3/WT) - ΔΔG(apo) (kcal/mol)*	*ΔΔΔG* class	z-score	Effect	Pred	Pred Conf	FGFR3_AA	PANEL
466		**7**			GLU	*LYS*	**2**	1.3569	1.5962	0.2393		1.05	No structural effects identified	SNP	0.7	466	**MUT_PANEL**
469		**4**			ARG	*GLN*	**1**	0.8761	3.2061	2.33	Destabilising - low	1.12	No structural effects identified	SNP	0.76	469	
490		**2**			GLU	*GLY*	**2**	1.4404	1.5994	0.159		-0.25	No structural effects identified	SNP	0.73	490	
500		**1**			ALA	*THR*	**1**	0.1759	-0.2716	-0.4475	Stabilising - low	-0.66	No structural effects identified	SNP	0.85	500	**MUT_PANEL**
505		**1**			VAL	*ILE*	**1**	1.1085	-0.2999	-1.4084	Stabilising - high	0.63	Conserved site	SNP	0.59	505	
507		**3**			VAL	*MET*	**2**	0.5384	5.6024	5.064	Destabilising - low	0.83	Clash|Conserved site	PD	0.8	507	
538	Molecular brake	**4**	Dysplasia & cancer mutation		ILE	*PHE*	**1**	11.33951	3.8555	-7.48401	Stabilising - very high	0.63	No structural effects identified	SNP	0.64	538	**MUT_PANEL**
					ILE	*VAL*	**0**	0.6259	1.0697	0.4438	Destabilising - low	-1.01	No structural effects identified	SNP	0	538	**MUT_PANEL**
540	Molecular brake	**61**	Dysplasia & cancer mutation		ASN	*LYS*	**0**	2.1699	-0.3467	-2.5166	Stabilising - very high	-1.36	HBonds|Conserved site	0	0	540	**MUT_PANEL**
					ASN	*SER*	**1**	1.8546	1.5002	-0.3544	Stabilising - low	-1.09	Conserved site	SNP	0.78	540	**MUT_PANEL**
555	Gatekeeper	**2**			VAL	*MET*	**1**	-0.3313	-0.7755	-0.4442	Stabilising - low	-1.83	Conserved site	SNP	0.07	555	**MUT_PANEL**
569		**1**			ALA	*VAL*	**1**	1.1687	1.3753	0.2066		-0.32	No structural effects identified	SNP	0.87	569	
572		**1**			PRO	*ALA*	**0**	1.9	1.1313	-0.7687	Stabilising - medium	-0.57	No structural effects identified	SNP	0	572	**MUT_PANEL**
576		**2**			ASP	*ASN*	**1**	0.1427	-0.1713	-0.314		-0.32	No structural effects identified	SNP	0.82	576	
582		**4**			CYS	*PHE*	**2**	1.6788	-0.1992	-1.878	Stabilising - high	-0.61	Surface Phobic	SNP	0.76	582	**MUT_PANEL**
603		**6**			ARG	*GLN*	**3**	0.8708	0.6045	-0.2663		-1.25	Conserved site	SNP	0.78	603	
608		**2**			LEU	*MET*	**1**	0.7697	0.0215	-0.7482	Stabilising - medium	0.75	Conserved site		*0.04*	608	
614	Catalytic loop	**1**			ILE	*ASN*	**1**	2.463	3.82	1.357	Destabilising - low	1.90	Core Philic|Conserved site	PD	0.6	614	
616	Catalytic loop,HRD motif	**4**	Dysplasia & cancer mutation		ARG	*GLY*	**1**	0.6658	-0.4531	-1.1189	Stabilising - medium	0.58	Buried Charge|HBonds|Conserved site	PD	0.78	616	
617	Catalytic loop,HRD motif	**1**			ASP	*GLY*	**1**	2.5016	-0.2262	-2.7278	Stabilising - very high	2.32	Buried Charge|HBonds|SProtFT	PD	0.65	617	**MUT_PANEL**
621	Catalytic loop	**3**	Dysplasia & cancer mutation		ARG	*HIS*	**2**	-0.414	1.4743	1.8883	Destabilising - low	0.74	HBonds|Conserved site	SNP	0.02	621	
627		**6**			GLU	*ASP*	**1**	0.4188	0.4545	0.0357		-0.57	No structural effects identified	SNP	0.86	627	**MUT_PANEL**
					GLU	*GLY*	**1**	0.7871	0.9393	0.1522		-0.32	No structural effects identified	SNP	0.72	627	
					GLU	*LYS*	**2**	0.143	0.3906	0.2476		-0.77	No structural effects identified	SNP	0.79	627	
					GLU	*VAL*	**1**	0.6774	0.871	0.1936		0.45	Surface Phobic	SNP	0.73	627	
630		**2**			VAL	*ALA*	**1**	1.3942	1.4217	0.0275		-0.38	Conserved site	SNP	0.68	630	
					VAL	*MET*	**1**	0.2983	0.5522	0.2539		-0.29	Conserved site	SNP	0.7	630	**MUT_PANEL**
636	Activation loop,DFG motif,Regulatory spine	**1**			PHE	*LEU*	**1**	1.5435	2.679	1.1355	Destabilising - low	0.69	Conserved site	SNP	0.61	636	
637	Activation loop,DFG motif	**2**			GLY	*TRP*	**2**	14.60959	-1.777	-16.38659	Stabilising - very high	2.34	Conserved site	SNP	0.67	637	**MUT_PANEL**
640	Activation loop	**3**			ARG	*TRP*	**2**	-0.1865	2.9882	3.1747	Destabilising - low	2.48	Conserved site|Surface Phobic	PD	0.55	640	
641	Activation loop	**5**			ASP	*ASN*	**2**	0.264	0.3708	0.1068		-0.33	No structural effects identified	SNP	0.75	641	**MUT_PANEL**
					ASP	*GLY*	**1**	0.6006	1.6588	1.0582	Destabilising - low	-0.28	HBonds	SNP	0.55	641	**MUT_PANEL**
643	Activation loop	**4**			HIS	*ARG*	**3**	-0.2915	-1.3046	-1.0131	Stabilising - medium	-1.07	No structural effects identified	SNP	0.82	643	
					HIS	*ASP*	**1**	-0.2355	0.5107	0.7462	Destabilising - low	-1.78	No structural effects identified	SNP	0.81	643	**MUT_PANEL**
646	Activation loop	**7**			ASP	*ASN*	**1**	0.8063	0.3807	-0.4256	Stabilising - low	-0.91	Conserved site	SNP	0.82	646	
					ASP	*GLY*	**1**	2.2881	0.5843	-1.7038	Stabilising - high	-1.00	Conserved site	SNP	0.79	646	
					ASP	*TYR*	**1**	1.3938	-0.0788	-1.4726	Stabilising - high	-0.28	Conserved site	SNP	0.81	646	**MUT_PANEL**
647	Activation loop,Phosphorylated tyrosines	**0**			TYR	*CYS*	**0**	2.0028	2.2561	0.2533		0.13	Conserved site	0	0	647	**MUT_PANEL**
650	Activation loop	**210**	Dysplasia & cancer mutation		LYS	*ASN*	**4**	0.2716	-2.504	-2.7756	Stabilising - very high	-0.28	Buried Charge	SNP	0.05	650	**MUT_PANEL**
					LYS	*GLN*	**6**	0.3504	-2.3932	-2.7436	Stabilising - very high	-0.65	Buried Charge	SNP	0.01	650	
					LYS	*GLU*	**83**	-0.0104	-4.2792	-4.2688	Stabilising - very high	-0.38	No structural effects identified	SNP	0.14	650	**MUT_PANEL**
					LYS	*MET*	**81**	-0.1739	-4.0829	-3.909	Stabilising - very high	-0.39	Buried Charge|HBonds	PD	0.6	650	
					LYS	*THR*	**4**	-0.1266	-1.2372	-1.1106	Stabilising - medium	-0.40	Buried Charge		*0.02*	650	
653	Activation loop	**2**			ASN	*HIS*	**1**	0.7217	0.1799	-0.5418	Stabilising - low	-1.05	Conserved site	SNP	0.84	653	**MUT_PANEL**
					ASN	*SER*	**1**	0.2161	0.0724	-0.1437		-1.63	Conserved site	SNP	0.84	653	
669		**5**	Dysplasia & cancer mutation		ARG	*GLN*	**1**	-0.7112	-0.5591	0.1521		-1.10	No structural effects identified	SNP	0.79	669	**MUT_PANEL**
					ARG	*GLY*	**0**	-0.319	-0.164	0.155		-1.78	No structural effects identified	SNP	0	669	**MUT_PANEL**
677		**4**			VAL	*ILE*	**1**	-0.3179	-0.4012	-0.0833		-0.44	Conserved site	SNP	0.6	677	**MUT_PANEL**
679		**2**			SER	*PHE*	**1**	4.4703	2.753	-1.7173	Stabilising - high	2.10	HBonds|Conserved site	PD	0.72	679	
686		**4**			GLU	*LYS*	**2**	5.2432	4.6084	-0.6348	Stabilising - low	2.05	Buried Charge|HBonds|Conserved site	PD	0.73	686	
689		**1**			THR	*MET*	**1**	-1.5705	-0.47	1.1005	Destabilising - low	0.79	HBonds	SNP	0.58	689	
696		**1**			PRO	*LEU*	**1**	1.3335	1.5784	0.2449		0.44	Surface Phobic	SNP	0.83	696	
697		**47**			GLY	*CYS*	**44**	2.0335	3.5217	1.4882	Destabilising - low	0.53	Conserved site	SNP	0.65	697	**MUT_PANEL**
700		**1**			VAL	*ALA*	**0**	0.7684	0.7455	-0.0229		-1.45	No structural effects identified	SNP	0.84	700	
715		**2**			LYS	*MET*	**2**	0.6027	0.0987	-0.504	Stabilising - low	-0.29	HBonds|Conserved site	SNP	0.58	715	
716		**4**			PRO	*HIS*	**3**	37.7316	27.0001	-10.7315	Stabilising - very high	1.36	Buried Charge|Core Philic	PD	0.73	716	
725		**1**			MET	*ILE*	**1**	0.3884	0.3385	-0.0499		-0.64	No structural effects identified	SNP	0.8	725	

Summary of bioinformatics analyses for FGFR3 kinase-domain for all residues having at least 1 cancer mutation (plus panel mutant Y647C)

Molecular Brake residues (538 & 540) are part of a cluster of mutations (highlighted in light blue) that encompass the pharmacologically important gatekeeper (V555M). Mutations I538F, D540K/S & V555M all show evidence for shifting equilibrium to favour active kinase conformation according to FOLDX.

Observed oncogenic mutations in A-loop residues are predicted to preferentially stabilise active kinase conformation in 60% of cases (where they don't also involve pathogenic mutations according to Condel or SAAP) and show pronounced stabilisation effects for the K650 hotspot.

Activating mutations R669G/Q are in a mutation cluster with A-loop residues.

"Hotspots" are highlighted red where 10 or more cancer mutations are observed. Condel scores: red (>Q(uartile)4), mid-red (>Q3)

FOLDX ΔΔΔG classified for each mutant using percentiles calculated from the total distribution of values for all possible mutants (5%: Stabilising - very high, 10%: Stabilising - high, 20%: Stabilising - medium and 30%: Stabilising - low; destabilising mutants were categorised equivalently)

FOLDX equilibrium predictions (excluding pathogenic mutations E466, D617 & G637 and based on ~7 fold activating mutations in Figure 2A including I538V; using FOLDX very high and high only):

Sensitivity (True Positive Rate) = TP/(TP+FN) = 0.43; Positive Predictive Value = TP/(TP+FP) = 0.5

Specificity (True Negative Rate) = TN/(TN+FP) = 0.63; Negative Predictive Value = TN/(TN+FN) = 0.75
