# Supplementary material for: Landscape of activating cancer mutations in FGFR kinases and their differential responses to inhibitors in clinical use
Source: Oncotarget. 2016 Mar 16;7(17):24252–68. doi: 10.18632/oncotarget.8132 (PMC5029699; doi:10.18632/oncotarget.8132)
Supplement: Supplementary file 3 [file oncotarget-07-24252-s003.pdf]

Supplemental Table S1b: Summary of Bioinformatics Predictions of SNV Effects.

Panel mutations			Bioinformatics methods indicating SNV effect						
			Sequence based			Seq & struct	Structure-based		
			Observed mutations		Predicted	consequence	Clustering	Stabilising?	
Position	Native	Mutant	*Cancer & Dysplasia*	*F1-4 high freq cancer*	*Condel*	*SAAP/pred*	*MutClust*	*FOLDX*	Total effects
466	GLU	*LYS*			■				1
500	ALA	*THR*							0
538	ILE	*PHE*	■				■	■	**3**
538	ILE	*VAL*	■				■		2
540	ASN	*LYS*	■	■		■	■	■	5
540	ASN	*SER*	■			■	■		**3**
555	VAL	*MET*				■	■		**2**
572	PRO	*ALA*							0
582	CYS	*PHE*				■		■	2
617	ASP	*GLY*			■	■		■	**3**
627	GLU	*ASP*							0
630	VAL	*MET*				■			1
637	GLY	*TRP*			■	■		■	**3**
641	ASP	*ASN*					■		1
641	ASP	*GLY*				■	■		2
643	HIS	*ASP*							0
646	ASP	*TYR*				■	■	■	**3**
647	TYR	*CYS*				■			1
650	LYS	*ASN*	■			■	■	■	**4**
650	LYS	*GLU*	■	■			■	■	**4**
653	ASN	*HIS*				■			1
669	ARG	*GLN*	■				■		2
669	ARG	*GLY*	■				■		2
677	VAL	*ILE*				■			1
697	GLY	*CYS*		■		■			2

FOLDX stabilising: Very High and High only (top 10% most stabilising)
