# Supplementary material for: Landscape of activating cancer mutations in FGFR kinases and their differential responses to inhibitors in clinical use
Source: Oncotarget. 2016 Mar 16;7(17):24252–68. doi: 10.18632/oncotarget.8132 (PMC5029699; doi:10.18632/oncotarget.8132)
Supplement: Supplementary file 4 [file oncotarget-07-24252-s004.pdf]

Supplemental Table S1c: Summary of Overlap between Experimental and Computational Predictions.

Panel mutations			Evidence of activating mutation			
			Experimental	Computational		Other effects
Position	Native	Mutant		Postive pred	Negative pred	
466	GLU	*LYS*	N	Condel/PD		
500	ALA	*THR*	N			
538	ILE	*PHE*	N		FOLDX + MutClust	
538	ILE	*VAL*	**Y**	MutClust		
540	ASN	*LYS*	**Y**	FOLDX + MutClust		SAAP:H-Bonds/conserved
540	ASN	*SER*	**Y**	MutClust		SAAP:Conserved
555	VAL	*MET*	**Part**	MutClust		SAAP:Conserved
572	PRO	*ALA*	N			
582	CYS	*PHE*	N		FOLDX	SAAP:surface-phobic
617	ASP	*GLY*	N	Condel/PD		SAAP/PD
627	GLU	*ASP*	N			
630	VAL	*MET*	N			SAAP:Conserved
637	GLY	*TRP*	N	Condel/PD		
641	ASP	*ASN*	**Part**	MutClust		
641	ASP	*GLY*	**Part**	MutClust		SAAP:H-Bonds
643	HIS	*ASP*	N			
646	ASP	*TYR*	N		FOLDX + MutClust	SAAP:Conserved
647	TYR	*CYS*	N			SAAP:Conserved
650	LYS	*ASN*	**Y**	FOLDX + MutClust		SAAP:buried charge
650	LYS	*GLU*	**Y**	FOLDX + MutClust		
653	ASN	*HIS*	N			SAAP:Conserved
669	ARG	*GLN*	**Y**	MutClust		
669	ARG	*GLY*	**Y**	MutClust		
677	VAL	*ILE*	N			SAAP:Conserved
697	GLY	*CYS*	N			SAAP:Conserved

Experimental: "Y"- activating according to Figure 2A (7 fold); "Part" ~5 fold
FOLDX stabilising: Very High and High only (top 10% most stabilising)
Condel/PD: Condel pathogenic deleterious
