# Supplementary material for: Landscape of activating cancer mutations in FGFR kinases and their differential responses to inhibitors in clinical use
Source: Oncotarget. 2016 Mar 16;7(17):24252–68. doi: 10.18632/oncotarget.8132 (PMC5029699; doi:10.18632/oncotarget.8132)
Supplement: Supplementary file 5 [file oncotarget-07-24252-s005.pdf]

Supplemental Table S2: X-ray data collection and refinement statistics.

	**FGFR1 R675G**	**FGFR1 bound to JNJ42756493**
Space group	$P\ 2_1\ 2_1\ 2_1$	C2
Cell dimensions	a = 76.3 Å; b = 152.3 Å; c = 195.9 Å; $\alpha = \beta = \gamma = 90^{o}$	a = 209.1 Å; b = 57.9 Å; c = 65.3 Å; $\alpha = 90^{o}$; $\beta = 107.5^{o}$; $\gamma = 90^{o}$
Resolution range (Å)	76.26 – 2.58	62.24 – 1.67
R_{symm}[a] (outer shell)	0.097 (0.603)	0.045 (1.027)
$I/\sigma I$ (outer shell)	10.4 (1.5)	20.3 (1.5)
Completeness (outer shell) %	98.2 (97.2)	96.7 (75.5)
Total number of reflections	288,249	577657
Number of unique reflections	70,919	89791
Redundancy (outer shell)	4.1 (3.1)	6.4 (4.8)
Wilson B-factor (Å^2)	56.0	29.90
R_{cryst}[b]/R_{free}[c]	0.194/0.255	0.184/0.209
Average B-factor (Å^2)		
Overall	52.8	37.32
Protein (chain A, B, C, D, E)	53.8, 45.0, 50.5, 45.1, 71.7	33.12, 40.10
Solvent	47.2	45.8
Ligands (SO_4^{2-}, PEG, Cl^-, $CH_3CO_2^-$)	88.1, 65.1, 60.3, 68.5	28.16, 46.60
RMS deviation		
Bond length (Å)	0.003	0.010
Bond angle (°)	0.752	0.97
Ramachandran plot statistics		
Favoured (%)	94.5	98.6
Less favoured (%)	5.5	1.4
PDB ID	**5FLF**	**5EW8**

[a]$R_{symm} = \Sigma_h \Sigma_i |I(h) - I_i(h)| / \Sigma_h \Sigma_i I_i(h)$, where $I_i(h)$ and $I(h)$ are the ith and the mean measurements of the intensity of reflection h, respectively.

[b]$R_{cryst} = \Sigma_h |F_o - F_c| / \Sigma_h F_o$, where F_o and F_c are the observed and calculated structure factor amplitudes of reflection h, respectively.

[c]R_{free} is equal to R_{cryst} for a randomly selected 5.0% subset of reflections not used in the refinement.
