# Supplementary material for: Landscape of activating cancer mutations in FGFR kinases and their differential responses to inhibitors in clinical use
Source: Oncotarget. 2016 Mar 16;7(17):24252–68. doi: 10.18632/oncotarget.8132 (PMC5029699; doi:10.18632/oncotarget.8132)
Supplement: Supplementary file 6 [file oncotarget-07-24252-s006.pdf]

Supplemental Table S3. Measurements of Ki for selected FGFR3 variants and inhibitors

FGFR3	K_i of FGFR Inhibitor / nM				
	**AZD4547**	**BGJ-398**	**TKI258**	**JNJ42756493**	**AP24534**
**WT**	4.4 ± 2.4	69.3 ± 7.1	65.4 ± 14.6	1.87 ± 0.24	94.5 ± 12.2
**V555M**	82.5 ± 10.9	511 ± 40	12.9 ± 3.8	330 ± 81	184.5 ± 18.9
**K650E**	14.3 ± 1.3	14.0 ± 2.7	41.7 ± 2.5	3.93 ± 1.38	127 ± 15
**R669G**	26.3 ± 7.0	12.9 ± 3.5	341 ± 59	6.7 ± 1.4	69.9 ± 14.0
**N540S**	18.7 ± 2.2	45 ± 14	294 ± 42	35.2 ± 11.0	41.9 ± 4.0
**N540K**	99.7 ± 9.8	13.6 ± 1.7	124 ± 17	9.06 ± 0.8	75.9 ± 14.3
**I538V**	152.6 ± 1.7	236.8 ± 78.1	1604 ± 6.5	72.0 ± 7.8	674.2 ± 99.7
